# Data Products, Quality and Validation of the DLR Earth Sensing Imaging Spectrometer (DESIS)

**DOI:** 10.3390/s19204471

**Published:** 2019-10-15

**Authors:** Kevin Alonso, Martin Bachmann, Kara Burch, Emiliano Carmona, Daniele Cerra, Raquel de los Reyes, Daniele Dietrich, Uta Heiden, Andreas Hölderlin, Jack Ickes, Uwe Knodt, David Krutz, Heath Lester, Rupert Müller, Mary Pagnutti, Peter Reinartz, Rudolf Richter, Robert Ryan, Ilse Sebastian, Mirco Tegler

**Affiliations:** 1 Remote Sensing Technology Institute, DLR, Oberpfaffenhofen, 82234 Weßling, Germany; Kevin.AlonsoGonzalez@dlr.de (K.A.); Emiliano.Carmona@dlr.de (E.C.); Daniele.Cerra@dlr.de (D.C.); Raquel.delosReyes@dlr.de (R.d.l.R.); Peter.Reinartz@dlr.de (P.R.); Rudolf.Richter@dlr.de (R.R.); 2 German Remote Sensing Data Center, DLR, Oberpfaffenhofen, 82234 Weßling, Germany; Martin.Bachmann@dlr.de (M.B.); Daniele.Dietrich@dlr.de (D.D.); Uta.Heiden@dlr.de (U.H.); Mirco.Tegler@dlr.de (M.T.); 3 Innovative Imaging and Research, Corp. (I2R), Building 1103, Suite 140C, Stennis Space Center, Hancock County, MS 39529, USA; kburch@i2rcorp.com (K.B.); mpagnutti@i2rcorp.com (M.P.); rryan@i2rcorp.com (R.R.); 4 Technology Marketing, DLR, Linder Höhe, 51147 Köln, Germany; Andreas.Hoelderlin@dlr.de; 5 Teledyne Brown Engineering (TBE), 300 Sparkman Drive, Huntsville, AL 35805, USA; jack.ickes@Teledyne.com (J.I.); Heath.Lester@Teledyne.com (H.L.); 6 Strategic services, DLR, Linder Höhe, 51147 Köln, Germany; Uwe.Knodt@dlr.de; 7 Institute of Optical Sensor Systems, DLR, Rutherfordstraße 2, 12489 Berlin, Germany; David.Krutz@dlr.de (D.K.); Ilse.Sebastian@dlr.de (I.S.)

**Keywords:** hyperspectral remote sensing, imaging spectrometry, earth observation, DESIS, MUSES, ISS

## Abstract

Imaging spectrometry from aerial or spaceborne platforms, also known as hyperspectral remote sensing, provides dense sampled and fine structured spectral information for each image pixel, allowing the user to identify and characterize Earth surface materials such as minerals in rocks and soils, vegetation types and stress indicators, and water constituents. The recently launched DLR Earth Sensing Imaging Spectrometer (DESIS) installed on the International Space Station (ISS) closes the long-term gap of sparsely available spaceborne imaging spectrometry data and will be part of the upcoming fleet of such new instruments in orbit. DESIS measures in the spectral range from 400 and 1000 nm with a spectral sampling distance of 2.55 nm and a Full Width Half Maximum (FWHM) of about 3.5 nm. The ground sample distance is 30 m with 1024 pixels across track. In this article, a detailed review is given on the applicability of DESIS data based on the specifics of the instrument, the characteristics of the ISS orbit, and the methods applied to generate products. The various DESIS data products available for users are described with the focus on specific processing steps. The results of the data quality and product validation studies show that top-of-atmosphere radiance, geometrically corrected, and bottom-of-atmosphere reflectance products meet the mission requirements. The limitations of the DESIS data products are also subject to a critical examination.

## 1. Introduction

Airborne and spaceborne imaging spectrometers have advanced our understanding of the dynamic processes of ecosystems by enabling quantification of geochemical, biochemical and biophysical characteristics of the Earth through collection of contiguous spectra of the Earth’s surface over a defined wavelength range for each image pixel. The fine spectral resolution (typically in the range of 2–20 nm) of imaging spectrometers has several advantages compared to multispectral instruments that have typically 10–20 spectral bands with bandwidths of 50–200 nm and large gaps between these broad bands. In principle, optical Earth observing instruments record the interaction of solar irradiation with the Earth’s surface and atmosphere. Imaging spectrometers allow for the quantitative measurement of the photon–matter interactions with very high spectral detail and accuracy [1]. With this, we can: (i) identify Earth surface materials such as minerals [2], vegetation invasive species [3] and urban surfaces [4]; (ii) quantify biochemical parameter such as the chlorophyll content of vegetation stands [5,6], soil organic matter [7,8] and water quality [9]; (iii) identify the background signal shadowing effects in urban areas [10], soil background for agricultural fields or vice versa [11] with unmixing techniques; and (iv) directly measure the composition of the intervening atmosphere to calculate precise surface reflectance [12] using radiative transfer approaches.

Operational spaceborne imaging spectrometer systems with sufficient spectral and spatial resolution, high signal-to-noise ratio (SNR) and good revisit times are still a challenge. This is due to the required trade-off between spectral bandwidth and the required energy (radiation) to illuminate detector elements (spatial resolution) to keep a sufficient signal to noise ratio (data quality). There are several categories of spaceborne imaging spectrometer missions in operation and/or in development. The first are operating missions such as CHRIS on PROBA-1 [13], Chinese Tiangong-1 [14,15], the Indian Hyperspectral Imaging Satellite (HySIS) (weblink available) and the Italian PRISMA mission [16], as well as future missions such as the German EnMAP [17], the Israeli SHALOM mission (REF) and ESA’s FLEX mission [18], which are technology demonstrators as well as science missions to prepare for more advanced spaceborne imaging spectrometers and suitable analysis techniques. Another category includes large operational mapping missions such as the Copernicus Hyperspectral Imaging Mission for the Environment [19] and NASA’s Surface Biology and Geology mission (SBG) [20], which have the objective of providing global coverage at high temporal resolution to boost operational product generation and the commercial use of data to support economic growth. A third category comprises the various initiatives of cubesat constellations with imaging spectrometers on board, including the operating HyperSCOUT mission [21] and the planned CSIMBA mission [22]. Such missions investigate their capability to reduce revisit time and save high mission development costs, but also to test onboard data processing for the development of near-real-time products from spaceborne imaging spectrometers.

In the near future, the International Space Station (ISS) will host several imaging spectrometers such as the upcoming NASA Earth Surface Mineral Dust Source Investigation mission and the Japanese HISUI imager [23]. Additionally, NASA’s Climate Absolute Radiance and Refractivity Observatory (CLARREO) Pathfinder mission [24] is in development to enable high accuracy SI-Traceable calibration for various Earth observing missions. DLR’s Earth Sensing Imaging Spectrometer (DESIS), jointly developed by the German Aerospace Center (DLR) and Teledyne Brown Engineering, is the first operating imaging spectrometer onboard the ISS [25,26]. DESIS is integrated into the Multi-User System for Earth Sensing (MUSES) platform, which provides accommodations for two large and two small hosted payloads and provides position and attitude measurements, master time, data downlink, and other core services common for each payload [27]. DESIS records data with an exceptionally high spectral resolution of 2.55 nm and a spatial resolution on the ground of about 30 m pixel size. In addition, DESIS is opening up further special fields of application. The off-nadir capability of DESIS with $\pm 15^{\circ }$ along the track during one image strip acquisition enables investigations of multiangular characteristics of objects on Earth, providing additional target-specific signatures. Finally, DESIS can be seen as a precursor instrument for EnMAP, as it has the same detectors in the VNIR range [17,26]. The experience gained, especially in the laboratory calibration and commissioning phase of DESIS, will be incorporated into the EnMAP program.

This article does not describe the design or specifications of the DESIS instrument and the MUSES platform, but rather the data products available to users and their quality as determined during the commissioning phase. For the DESIS instrument design, please refer to [26] and for the MUSES platform please refer to [27]. The article is organized as follows. In Section 2, a review of possible hyperspectral applications with focus on the DESIS mission and the ISS orbit characteristics is given. An overview of available products and their specific processing is described in Section 3. In Section 4, the validation results of the commissioning phase of DESIS are presented with the purpose of enabling users to assess the quality of the data. The limitations of the data are given in Section 5. Finally, Section 6 describes a data fusion experiment of DESIS data with Sentinel-2 data and demonstrates the potential of multi-modal applications, especially if other Earth sensing instruments are placed in the MUSES platform.

## 2. Application Fields of DESIS

Imaging spectroscopy data opens up new opportunities for Earth surface identification and quantification. The spectral sampling of DESIS of 2.55 nm across the VNIR spectral wavelength range combined with 30 m spatial resolution allows new developments for a wide range of applications. The following paragraphs emphasize potential applications where especially DESIS can play a vital role to identify surface materials and to quantify their abundance in a given pixel. However, these applications are not exclusive and other applications that are currently barely explored such as the use of DESIS data for urban areas [28] or for detecting rare Earth elements are not explicitly mentioned here.

### 2.1. Coastal and Inland Waters

Coastal and inland bodies of water are one of the most important and sensitive ecosystems worldwide. These zones host the most significant and diverse bacterial, algal, plant, and animal populations of the planet [29] because they supply food and freshwater; serve a key role in cycling of carbon, minerals and nutrients; and have far-reaching cultural and recreational impacts [30]. Coastal and inland waters are some of the most biologically diverse and at the same time endangered places on Earth [31]. Over 70 % of the human population lives near a coast, estuary, wetland, or coral reef. Due to the increasing eutrophication and pollution of inland and coastal water ecosystems, monitoring these areas with high spatial and spectral Earth Observation data is essential [32]. Several studies analyzed the satellite sensor requirements for a monitoring system resulting in 5–10 nm spectral resolution and a spatial resolution 17 m to 60 m pixel size, covering wavelength ranges up to 1000 nm [29,33,34,35]. Given these requirements, DESIS can play an essential role in developing a next-generation coastal and inland water monitoring system [36,37,38,39], allowing the retrieval of water reflectance, physical parameters such as turbidity and water clarity [40], suspended and dissolved water quality components (e.g., Chl-a concentration as a proxy of phytoplankton biomass), colored dissolved organic matter (CDOM) and total suspended matter (TSM) [9,41]. Compared to traditional EO systems based on MODIS, MERIS, Sentinel and Landsat [42,43], imaging spectroscopy has clear advantages in discriminating phytoplankton types [43], characterizing submerged habitat compositions [44], assessing water quality [32], observing environmental threats such as coral bleaching and estimating bathymetry [45].

### 2.2. Cryosphere

A relatively new field of application is using imaging spectroscopy data to interpret snow properties such as grain size [46], liquid water content, snow algae and light-absorbing impurities in snow and ice. Knowledge of these characteristics is important because the authors of [47,48] stated that the black carbon on snow surfaces has a much higher impact on snowmelt than the increase in air temperature. A better understanding of the influence of these properties on the dynamics of snow albedo can increase our understanding of water and energy fluxes in snow-covered areas and thus contributes to hydrological and climate models [49]. Although snow reflects mainly in the wavelength range 400–1400 nm, the major contribution can be found less than 1000 nm [50]. Thus, DESIS can contribute to the general understanding of the radiative forcing from dust (light-absorbing impurities in snow and ice, LAISI) and the dependency and complex feedback effects of the snow grain size. In general, LAISI leads to an increased absorption of solar radiation (radiative forcing) in the visible to near-infrared, a driver for melting snow in the mountains [51]. One of the feedback effects is grain coarsening, when water percolates vertically, that further decreases snow albedo. At the same time, smaller grains remaining on the surface mitigate this effect [52]. Recently, snow spectroscopy has been complemented by LIDAR data to support the derivation of the snow water equivalent, an important parameter to estimate freshwater quantities [53]. It should be therefore mentioned that ISS also carries a LIDAR instrument, Global Ecosystem Dynamics Investigation (GEDI) [54], that can be used to complement DESIS for characterizing the cryosphere.

### 2.3. Vegetation

An important field of application is the characterization of vegetation that comprises various topics such as species discrimination and invasive species detection; quantification of the structural, physiological, biochemical, or phenological traits of vegetation; and productivity and stress monitoring. Characterization of plant traits [55,56] gives insight into underlying ecophysiological processes of different vegetated environments [57] such as forests [58], agricultural areas [59] and natural vegetation stands [60]. Furthermore, vegetation traits are assimilated into complex decision support systems such as hydro-agroecological models [61], ecosystem process models to assess forest productivity [62,63] and forest biomass [64] or to assess biodiversity of natural areas [65]. The estimation of plant traits is based on diagnostic spectral features across the complete reflective range of 400–2500 nm. The DESIS wavelength range allows the direct quantification of vegetation pigments (e.g., chlorophyll, carotenoids, and anthocyanin) and the estimation of biophysical parameters (e.g., leaf area index and biomass). The quantification of nutrients, leaf and canopy water and biochemical constituents such as lignin and cellulose, which are important indicators of ecophysiological processes, is hampered by the missing SWIR wavelength range. However, in Huemmrich et al. [66] the potential of the observational ISS orbit characteristics for studying ecosystem carbon fluxes that can vary widely due to environmental conditions (e.g., irradiation and water supply) is described. Since the ISS orbit provides observations collected at different times of the day within a period of a few days, it can be used to study the seasonal and diurnal dynamics of ecosystem productivity, which would not be possible with Sentinel-2 and the Landsat fleet. It should be mentioned that the use of DESIS for vegetation monitoring would still be experimental, since ISS orbit characteristics also lead to longer periods in the vegetation period without any observation, which is different from Sentinel-2, which offers acquisitions every five days with comparable observation characteristics (e.g., quasi nadir observations and fixed equator crossing time), and thus contributes to the operational exploitation of these datasets. Nevertheless, DESIS as an imaging spectrometer holds the potential for an improved parameter retrieval for physical-based and empirical models [67,68] with reduced uncertainties in space and time [69] and it can boast novel approaches by interpreting spectrally detailed information that cannot be resolved by multispectral sensors [70].

### 2.4. Soil Sciences

Soils provide a large variety of ecosystem services, such as regulation of water and nutrient availability to support human food security and biodiversity.Soils are the largest terrestrial carbon pool on Earth [71]. Several soil regulations and policies, such as the EU Soil Thematic Strategy and Soil Framework Directory, express the demand to protect soils from overexploitation and degradation [72]. Imaging spectroscopy is a commonly used and accepted data source for soil spectral modeling and the quantification of soil constituents [73,74,75], which has resulted in mature data analysis packages such as the EnMAP Soil Mapper ENSOMAP [76] and the data mining engine PARACUDA [77]. These and other methodologies can support applications including the monitoring of soil status, soil fertility and soil threats [78] from space. The VNIR wavelength range is sensitive to iron oxides that are among the determinants of soil fertility and soil quality [79], as well as soil degradation [11,80]. Another important parameter to assess soil quality and fertility is soil organic carbon (SOC), which can be mapped and quantified by using the shape of the reflectance spectrum in the VNIR wavelength range [11,81,82,83]. However, it should be noted that soils are very complex systems and the correlation of a spectral feature to one soil property is not as straightforward as, for instance, SOC spectral features, and can be hidden or weakened by iron oxides [84,85]. Additionally, soil water content [85,86] and soil roughness [87,88] can affect the spectral reflectance of soils. The latter comprises the non-Lambertian behavior of soil surfaces due to irregularities and the effects from shadowed areas. The multiangular capabilities and the complex observation and illumination conditions of DESIS can support the analysis, and, ultimately, modeling of soil roughness effects from space.

### 2.5. Synergies

With the availability of multiple spaceborne imaging spectrometers in the coming years, it will be of great benefit to cross-calibrate these missions for multiple reasons. First, the end-user of hyperspectral datasets can achieve a higher coverage on ground in the spatial and temporal domains, especially since none of these missions is a global mapping mission. Next, there is the possibility to extend the spectral range of DESIS into the SWIR by combining multiple sensors, or the possibility to increase the spectral resolution within the VNIR spectral range thanks to the fine spectral resolution of DESIS. For all these synergistic activities, the shifting ISS orbit is a big benefit because matching observations with other EO satellite systems on other orbits are possible.

In this context, it is worth pointing out that, with the upcoming launch of HISUI, two additional imaging spectrometers will be mounted on the ISS, so that simultaneous data acquisitions from the same platforms will become possible, facilitating cross-validation and cross-calibration activities [89]. In addition, the spectrometers of the Climate Absolute Radiance and Refractivity Observatory (CLARREO) Pathfinder mission, which will be installed on the ISS around 2023 [24], will allow for new possibilities of highly accurate cross-calibration.

## 3. DESIS Products

The DESIS instrument can continuously collect data up to a maximum length of 3000 km on the ground. Such Earth data-takes are embedded with measurements of the dark signal before and after the acquisition. After a data screening process, which identifies and marks faulty or suspicious measurements, the data-take is divided into 1024 × 1024 pixel tiles, corresponding to 30 × 30 km${}^{2}$ on the ground. Together with derived metadata and orbit/attitude products, the tiles are placed in a long-term archive. Data of different processing levels can be requested via web portals (see [90]). Table 1 summarizes the products a user or customer can order.

The following sections give an overview of the DESIS products, as well as the specific algorithms applied. A detailed algorithm description and the product specifications can be found at the DLR DESIS Official Website [90]. This website also describes how users from the scientific community can obtain DESIS data and view the *License Agreement regarding the Use of the DESIS Data for Scientific Use*. For commercial applications, please visit [91]. It should be noted that the use of DESIS data with spectral sampling better than 10 nm requires a special approval (see also “Spectral Binning” in Section 3.1).

### 3.1. Top-of-Atmosphere Radiance Product (L1B)

The L1B processing algorithm is responsible for delivering at sensor Top-of-Atmosphere (TOA) radiance from the Digital Numbers (DN) obtained after read out of the focal plan array signal in the DESIS sensor. The DN to TOA radiance conversion can be described mathematically in a compact form:

(1) $$L=G\;\cdot \;(DN^{lin}-{\bar{DN}}^{dark})$$

where *L* is the TOA radiance at the sensor geometry, *G* is the radiometric correction factor, $DN^{lin}$ is the DESIS recorded digital value corrected for non-linearity, and ${\bar{DN}}^{dark}$ is the Dark Current (DC) correction computed from the DN values recorded with the shutter lid in closed position, which model the electronic noise of the instrument.

**Figure 1:**
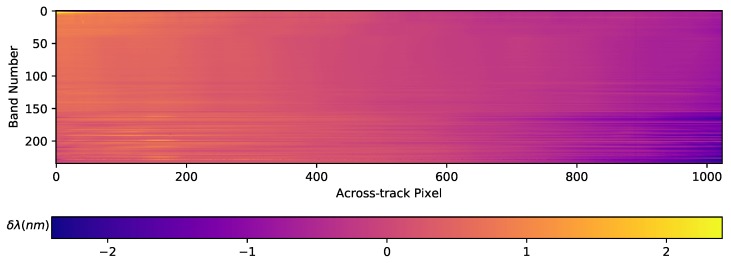
Quantification of the spectral smile on the DESIS focal plane as the difference of each detector element to the nominal band center wavelength. Lower bands are mostly dominated by a clocking effect between the grating plate and the focal plane. The NIR bands are also influenced by an etaloning effect, produced by reflections between sensor layers, leading to the appearance of interference patterns.

**Figure 2:**
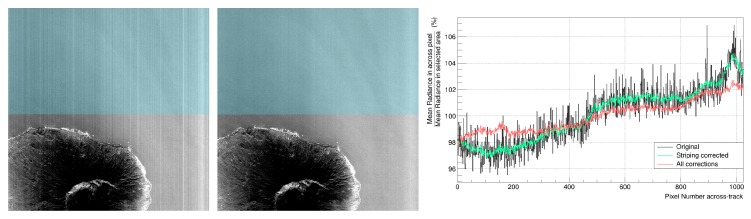
Correction of striping in DESIS Images: (**left**) original image band 16, with visible stripes (image stretched to emphasize the stripes); (**middle**) the same image after striping correction (same stretching); and (**right**) the relative variation of radiance across-track, averaged along-track over the colored area above the island. Colors in the right plot indicate the original image data (black), striping-corrected data (green) and data corrected by striping, flat-fielding and spectral-adjustment using the current DESIS calibration (red).

In addition to the radiometric conversion, the TOA radiance product is further processed in order to identify and reduce the influence of different effects, the most important being:

1. **Abnormal pixels**. For the detection of the abnormal pixels, along with the dead pixel map table, a scene-wise quality analysis is performed right after the radiometric conversion. The analysis results are part of the final TOA radiance product in the form of a quality quicklook, which describes the status of each pixel. Table 2 provides a list of the abnormal pixel types. Once the abnormal pixels are flagged, a hybrid interpolation method is used to minimize their impact in the final processing steps of the L1B processor. This hybrid interpolation selects the optimum value between spectral and spatial cubic spline interpolation. The selection criterion is based on the spectral gradient difference between the interpolated pixels and spatial neighbors.
2. **Rolling Shutter**. The DESIS sensor has a CMOS detector that uses a rolling shutter mode, which enables a higher frame rate and better SNR than a global shutter. The drawback of the rolling shutter is that each scan line (i.e., spectral channel) is collected at a slightly different time. Thus, each channel in a frame measures reflections from a different area on the ground due to the time delay between the beginnings of exposure for each of the spectral channels. The L1B processor accounts for the shift between the spectral channels within a frame and corrects it using bi-cubic spline interpolation on the along-track direction.
3. **Smile and Clocking**. The spectral smile effect results in a variation of the channel central wavelength in the across-track direction. Taking the measured central wavelength on the sensor’s center as the nominal central wavelength, the spectral smile produces a shift of the measured central wavelength with maximum values at the sensor edges. The TOA radiance product provides a smile corrected image by performing a bi-cubic interpolation over the spectral dimension on every pixel across-track. The difference between the nominal central wavelengths and the characterized wavelengths is shown on Figure 1. One of the main contributors to the mismatch is the clocking effect produced by a small misalignment between the grating plate and the focal plane. On the lower-half bands, this effect is contained within a 1 nm difference, excluding the values in the manufacturing defect region. For the upper-half bands, apart from the clocking, the optical etaloning effect increases the central wavelength mismatch. The etaloning effect appears on back-illuminated CCD sensor due to the transparency of silicon at NIR wavelengths. This property allows coherent light to reflect between the front and back surfaces producing interference patterns which disturb the measurements [92,93]. The influence of the etaloning is most noticeable on the highest bands and pixel values across-track.
4. **Striping**. Small pixel-to-pixel variations in the radiometric calibration factors of a push-broom sensor result in visible along-track stripes in the acquired images. These variations can be due to after-launch effects or sensor changes over time. A striping correction is introduced in the DESIS processing chain as a multiplicative correction on the smile-corrected radiometric values. Typically, the striping correction values observed in DESIS are below 1%, making it difficult to obtain the values from a simple update of the radiometric calibration tables. To address this effect, an iterative method, employing cubic splines to fit the across-track data using dozens of spatially homogeneous scenes and to find the parameters that minimize the stripes, has been implemented. An example of the striping effect on an image and its correction is shown in Figure 2.

Additionally, as a final processing step, the L1B processor is capable of performing binning operations along the spectral dimension, based on a user-configurable parameter.

5. **Spectral Binning**. DESIS processing chain supports four different spectral binning configurations. Binning ×1, or no-binning, is the nominal instrument data acquisition mode, offering 235 bands with a nominal spectral sampling of 2.55 nm and Full Width Half Maximum (FWHM) of 3.5 nm. Binning modes ×2, ×3, and ×4 provide spectral resolutions of 5.1 nm, 7.65 nm, and 10.2 nm, respectively. The strategy for the spectral binning follows the hardware read-out sequence. Thus, during processing, the bands are binned starting from the center of the focal plane towards the the edges.

### 3.2. Georeferenced and Resampled Product (L1C)

**Figure 3:**
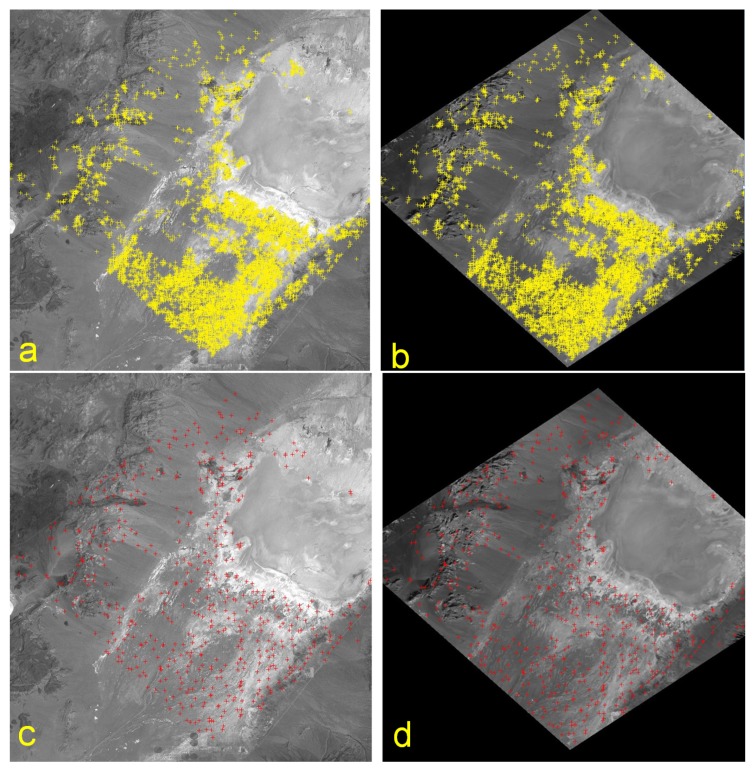
(First row) Homologous points (marked yellow) found by image matching. Left image (**a**): Reference (RI); Right image (**b**): coarsely orthorectified image by affine transformation (DESIS-PAN). (Second Row) Thinned out homologous points (marked red) between RI (**c**) and DESIS-PAN (**d**) based on best quality figure within a grid of 25 × 25 cells over the image. These points are used as GCPs to adjust the sensor model, whereas the remaining homologous points are used for quality assessment. Data: Railroad Valley, USA; 13 December 2018 18:23:11 UTC; $38.4467^{\circ }$ N $115.7512^{\circ }$ W; Sun Zenith $64.14^{\circ }$, Sun Azimuth $160.58^{\circ }$; Incident Angle: $0.8^{\circ }$.

Orthorectification is the process of generating map products by removing geometric distortions caused by the sensor internal geometry, the satellite motion during line-by-line data acquisition, and the terrain-related influences. The L1C processor generates orthoimages employing the rigorous technique of direct georeferencing [94,95]. The MUSES platform is equipped with a star tracker (sampling rate 10 Hz) and a miniature inertial measurement unit (sampling rate 50 Hz), providing a 10 Hz attitude measurement after filtering. The ISS GPS data provide position vectors and time tags at a sampling rate of 1 Hz, serving as a master time for the attitude system and the DESIS instrument with an accuracy of 0.25 ms (which is in terms of satellite movement about 1.8 m). The internal sensor geometry was characterized in the laboratory, where the main finding was that the consideration of an average keystone (i.e., averaged over the entire wavelength range) is sufficient for geometric processing. The maximum spectral deviations are less than 0.14 pixels. The user can choose the re-sampling method (nearest-neighbor, bilinear, and cubic convolution) and the map projection (UTM or geographic). To meet the accuracy requirement of better than 1 pixel in geolocation, existing global reference data with high geometric accuracy are used. The improvement consists of an on-the-fly image matching with the reference data to extract Ground Control Points (GCP) and adjust sensor model parameters. Currently, Landsat 7 ETM+ panchromatic global references with an 2D Root Mean Square Error (RMSE) of about 25 m [96] are used. Note that the RMSE values of the reference are strongly dependent on the region; for example, in North America, the RMSE is about 16 m, while, in Australia, about 30 m. The improvement of the geometric accuracy by Level 1C processing comprises the following steps:

1. Generate a panchromatic image (DESIS-PAN) using DESIS VNIR bands closest to the wavelengths of the reference image (RI), employing the global Landsat 7 ETM+ reference database.
2. Coarsely register the panchromatic DESIS-PAN by affine transformation based on current knowledge of the geometric mapping function.
3. Apply a Wallis filter to the DESIS-PAN and the RI to locally enhance the image contrast for a better image matching.
4. Perform a cascade of image matching methods to extract homologous points (see Figure 3).
  **BRISK:** Binary Robust Invariant Scalable Keypoints [97]
  **LLSQ:** Local Least Squares hierarchical intensity-based matching [98]
  **SIFT:** Scale-Invariant Feature Transform [99] (only used in case of a valid license)
5. After a highly selective outlier detection and removal, three-dimensional points are generated using the global SRTM 1 arcsec Digital Elevation Model (DEM) and split into Ground Control Points (GCP) for sensor model refinement and Control Points (CP) for geometric accuracy assessment (see [94]).
6. Within an iterative least squares estimation, the DESIS mounting angles are refined and applied for direct georeferencing. The least squares estimation includes a final outlier removal, where GCPs with the highest residual and greater than a threshold (here 2 pixels) are successively removed from the GCP set.

In the case image matching is not possible due to low textured image pairs (e.g., rain forest, deserts, and cloud and haze cover) or drastic changes in the land cover between the acquisition times of the reference image and the DESIS scene (e.g., agriculture fields and snow cover), geometric processing will rely only on the on-board position and attitude measurements, the laboratory geometric calibration, and the estimated boresight angles (instrument mounting angles with respect to the star tracker coordinate frame). This not only has an effect on the geolocation accuracy, but also on the atmospheric correction procedure, since taking into account the topography with a digital surface model (DSM) requires a co-registration accuracy of better than 1 pixel ( 30 m). Therefore, this function is automatically switched off for insufficiently aligned datasets, as radiometric artifacts would otherwise occur in the final product, especially in rough terrain.

### 3.3. Atmospheric Compensated Product (L2A)

The atmospheric compensation is performed by the L2A processor and corrects the data from atmospheric molecular absorption, scattering and aerosol effects. The software used is a tailored version of DLR’s PACO (Python Atmospheric COrrection, see [100]), which is based on ATCOR [101] and was independently validated for multispectral sensors [102].

**Figure 4:**
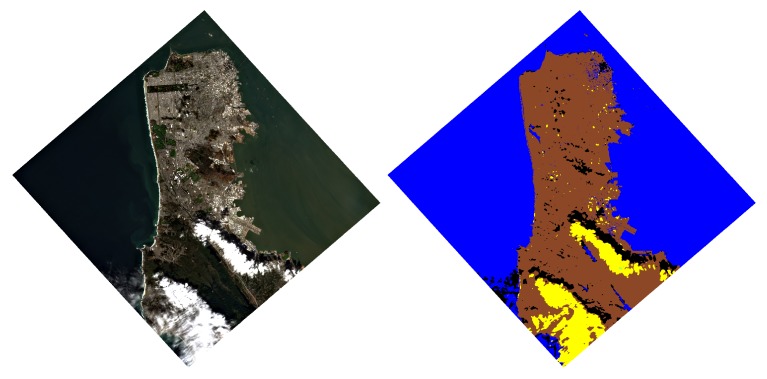
DESIS scene of San Francisco bay (10 October 2018): (**Left**) RGB map of the scene (R: 640 nm, G: 550 nm, B: 461 nm); and (**Right**) color composite of the masks relevant for this scene: land (2) in brown, water (8) in blue, shadows (1) in black and clouds (6 and 7) in yellow. The scene background is represented in white in both pictures.

The atmospheric correction (AC) can be performed considering the rugged terrain or approximating the surface to a flat-terrain of scene mean height. Both options are available to the user through the “TerrainCorrection” option. The atmospheric compensated products consist of:

- Bottom-of-atmosphere (BOA) surface reflectance in units ranging from 0 to 1.
- Quality masks containing classification, aerosol optical thickness (AOT) and water vapor (WV). The 10 layers (see Table 3) are ordered as follows, with the first eight indicating which pixels are classified according to the different criteria:

Figure 4 shows an example of the masks for a DESIS scene taken over San Francisco Bay area. This set of masks is very basic and can be used to extract more complete masks. The bit in the different layers can be used with simple logic algorithms to combine the information. For example, the total amount of water pixels in the scene will require the “or” logic combination of Layers 5, 7 and 8.

**Figure 5:**
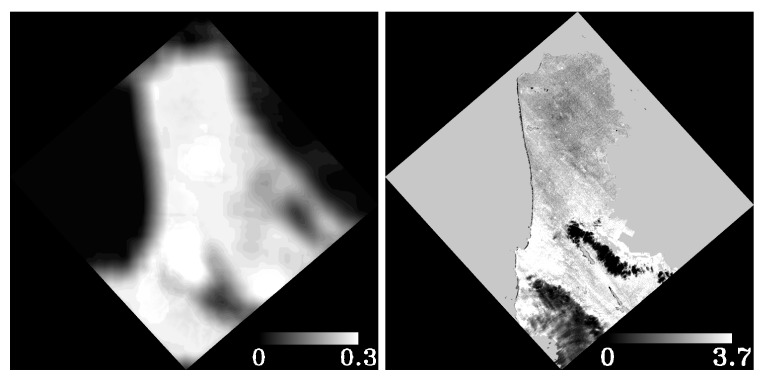
DESIS scene of San Francisco bay (10 October 2018): (**Left**) aerosol optical thickness map (Mask Layer 9); and (**Right**) water vapor map (Mask Layer 10). The maximum value in the plots corresponds to an AOT value of 0.26 and WV = 3.7 cm.

The last two layers: AOT and WV, are shown in Figure 5.

If the rugged terrain AC [101] option is selected, the scene’s corresponding Digital Elevation Model is calculated from the reference database (SRTM, 1 arcsec) (see Section 3.2).

The atmospheric correction performs the following steps and corresponding algorithms:

- High spectral resolution (0.4 μm) radiative transfer (RT) functions LUTs are simulated using MODTRAN (version 5.4.0) [103] for both mid-latitude summer and winter seasons.
- The simulated radiative transfer functions are transformed to sensor specific radiative transfer LUTs by convolving them with the sensor response function per band. The same response functions are used to calculate the solar irradiance for DESIS sensors using the Fontenla [104] solar model. The sensor response functions, RT LUTs and solar irradiance values are different for the different binning modes (Section 4.3.2).
- The scene’s corresponding season is automatically determined from the land surface temperature (LST) corresponding to the scene, with a season temperature threshold of 8 ${}^{\circ }$C (below which winter is assumed). The LST is retrieved from MODIS (Moderate Resolution Imaging Spectroradiometer) products, by querying the MODIS database MOD11C3 (version 6) [105], which contains the worldwide monthly averaged LST in a 0.05 degree grid.
- Masking: According to a set of pre-established thresholds, the pixels are classified into clouds, shadows, dark-dense vegetation (DDV), water, haze, etc.
- Aerosol Optical Thickness over land is retrieved per pixel using red and NIR surface reflectance of dark dense vegetation pixels identified within the scene [106].
- A water vapor map is calculated for each pixel with the Atmospheric Pre-corrected Differential Absorption (APDA) algorithm [107] using the water absorption region around 820 nm, interpolating several bands.
- Rugged-terrain [101] or flat-terrain Bottom-Of-Atmosphere reflectance: If less than 1% of the scene contains pixels with slopes $>6^{\circ }$, the flat-terrain scenario is assumed to retrieve the surface reflectance in order to avoid potential DEM artifacts.

## 4. Product Quality and Validation

During the commissioning phase, the quality of the data was evaluated and the products validated. Adjustments were made in an iterative process to ensure reproducible physical measurements. In particular, the radiometric and spectral characterization of the instrument was significantly improved by vicarious calibration using ground measurements at well known test sites and through cross-calibration with Landsat-8 and Sentinel-2 data. The following gives an overview of the data validation studies.

### 4.1. Temperature Monitoring and Dark Signal Stability

To ensure DESIS sensor stability, two main parameters are monitored: (i) the temperature of the sensor; and (ii) the dark signal or electronic noise.

**Figure 6:**
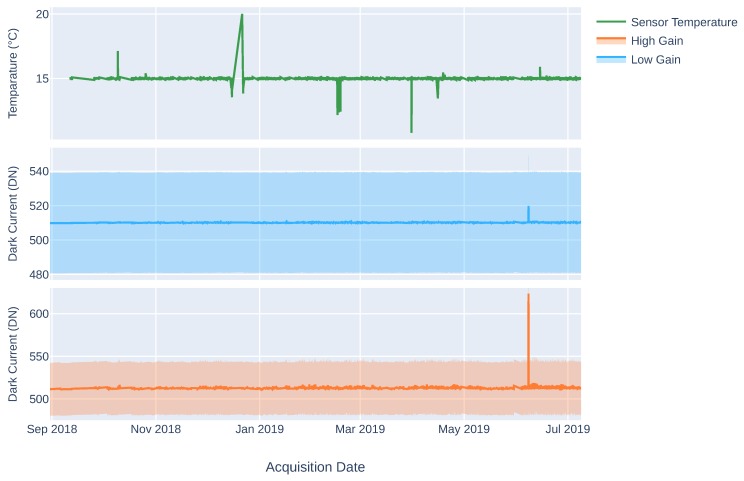
DESIS sensor historical monitoring results: (**Top**) historical temperatures of the focal plane; and (**middle**,**bottom**) historical DC values averaged for the whole focal plane and for a gain factor ×2 and ×10, respectively.

The sensor temperature is thermo-stabilized to 15 ${}^{\circ }C$. The first representation in Figure 6 shows the historic temperature values since the mission started. Deviations from the stabilized temperature due to exceptional circumstances do not show any impact on the Dark Current (DC) measurements. DC measurements are performed before and after each earth or calibration data-take. This strategy ensures a good characterization of the sensor’s electronic noise, which is subtracted from the data-take during the systematic corrections (see Section 3.1). The last two plots in Figure 6 show the averaged DC reference values over the focal plane for the 2 different available gain factors, low gain (×2) and high gain (×10).

Even though the aforementioned strategy is robust against dark signal variations up to a certain point, a stable dark signal behavior is desired. The results are fairly stable during the mission lifetime. Using the low gain (×2), the mean DC for the whole focal plane is 509.68 DN, with a standard deviation among pixels of 29.01 DN. In the high gain (×10) case, the mean value is 512.32 DN, with a standard deviation of 30.8 DN. The averaged standard deviation of the DC measurements is under 1 DN for both gains. On June 7, a spike on the DC measurements was recorded that was produced by a single non-nominal sensor behavior, which was solved returning the instrument behavior to normal.

**Figure 7:**
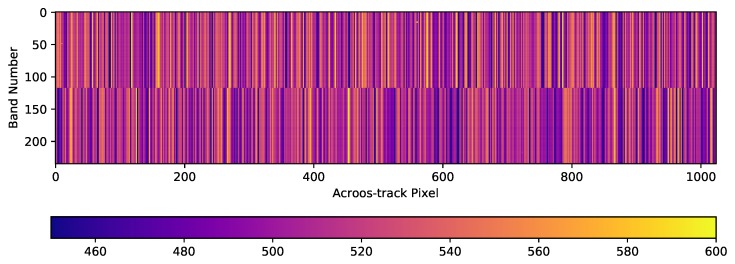
Historical averaged dark current values.

Performing a pixel level analysis, Figure 7 shows the historical averaged values per pixel of the whole focal plane. The two different data readout electronics divide the sensor in two, starting the data acquisition from the center bands and continuing to the spectral edges. The visible pattern is due to the readout configuration of the sensor. The two vertical halves of the sensor are read separately and all vertical pixels within each half of the sensor share the same readout electronics.

### 4.2. Radiometric Calibration and Properties

Most spaceborne optical instruments take advantage of on-board calibration assemblies (OBCA) such as solar diffusers, integrating spheres or focal plane LEDs (e.g., [17]). In addition, vicarious calibration approaches exist (e.g., [108]) that are used in conjunction with the OBCA measurements, or as the sole calibration reference (e.g., [109]). No solar diffusers or integrating spheres are available for DESIS, so the radiometric calibration is based on the pre-launch characterization of the instrument combined with the use of LED OBCA unit and vicarious calibration. The OBCA unit, described in [26], is mainly employed for monitoring the stability of the radiometric and spectral instrument response. In addition, it can be used to update the calibration parameters, but the non-uniform illumination of the focal plane introduces difficulties that have been avoided through the use of vicarious calibration. The vicarious calibration of DESIS uses suitable spectrally homogeneous scenes (including CEOS PICS and RadCalNet sites, see [108,110]), as well as cross-calibration using near-coincident Landsat-8 and Sentinel-2 scenes. Based on these datasets, an update of the existing radiometric coefficients (i.e., the relative and absolute radiometric calibration) and minor updates of the spectral characterization of the sensor were performed.

This update of the calibration was performed during the Commissioning Phase and currently represents the baseline calibration of the DESIS instrument. In the following sections, the two approaches used for the validation of the DESIS calibrated data are briefly described and the relevant findings are presented in Section 4.2.1 and Section 4.2.2, while Section 4.2.3 presents an evaluation of the DESIS Signal-to-Noise Ratio.

#### 4.2.1. Top-of-Atmosphere Validation against RadCalNet

Thus far, no publicly available radiometric reference at TOA-level exists. The Committee on Earth Observation Satellites (CEOS) set up an infrastructure which aims at providing SI-traceable reference measurements for post-launch radiometric calibration and validation. The RadCalNet [110] currently consists of four automated ground instrumentation sites, providing BOA reflectance and upscaled TOA reflectance estimates at 10 nm spectral resolution between 380 nm and 2500 nm. The sites are mostly non-vegetated and in semi-arid to arid regions, which are also spectrally smooth over a large spatial region. It is worth noting that, even though data should be provided for each day at 30 minutes intervals, a strict quality control process is used, so that only high-quality data are made available, which reduces the effective number of available datasets.

The following sites and dates used for the validation of the DESIS radiometric data properties, covering a time frame of six months, is shown (see Table 4).

As the TOA reflectance data from RadCalNet and the DESIS TOA radiance data are not directly comparable, the following processing steps are carried out.

The DESIS dataset is processed up to L1B using the standard processing scheme, so that it is available with a spectral resolution of ∼2.5 nm and in TOA radiances in units of (mW·cm${}^{-2}$·sr${}^{-1}$·μm${}^{-1}$). By using the geocoded shape file provided by RadCalNet, and linking this to the L1B geometry, the mean radiance ${\bar{L}}_{TOA,DESIS}$ within this area is derived.

Next, the RadCalNet dataset closest in time to the DESIS acquisition is selected. Then, the RadCalNet TOA reflectance $\rho_{TOA}$ and related uncertainties are converted to TOA radiance using the following equation:

(2) $$L_{TOA,RadCalNet}=\frac{\rho_{TOA,RadCalNet}\;\cos\left(SZA\right)\;E0}{\pi \;d^{2}}$$

with *d* being the Earth–sun distance in astronomical units, and E0 being the solar spectral irradiance model from Thuillier [111], endorsed by and available at CEOS IVOS [112], and SZA is the solar zenith angle at the time of acquisition. Finally, the ${\bar{L}}_{TOA,DESIS}$ is resampled to the coarser spectral resolution of $L_{TOA,RadCalNet}$ using the nominal center wavelengths, and converted to the proper radiance units.

**Figure 11:**
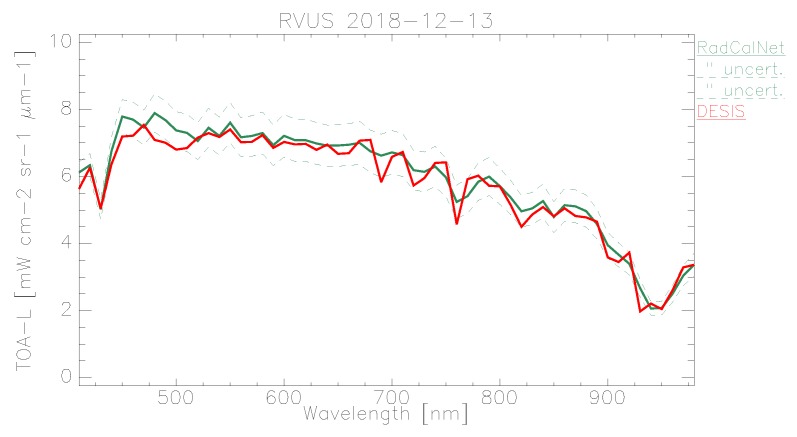
Comparison of RadCalNet TOA-L to DESIS mean TOA-L for RadCalNet location, RVUS, 13 December 2018, resampled to RadCalNet spectral resolution.

**Figure 12:**
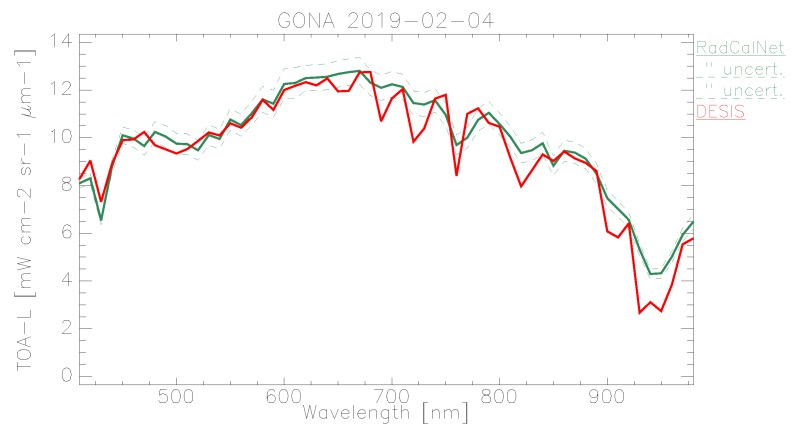
Comparison of RadCalNet TOA-L to DESIS mean TOA-L for RadCalNet location, GONA, 4 February 2019, resampled to RadCalNet spectral resolution.

**Figure 13:**
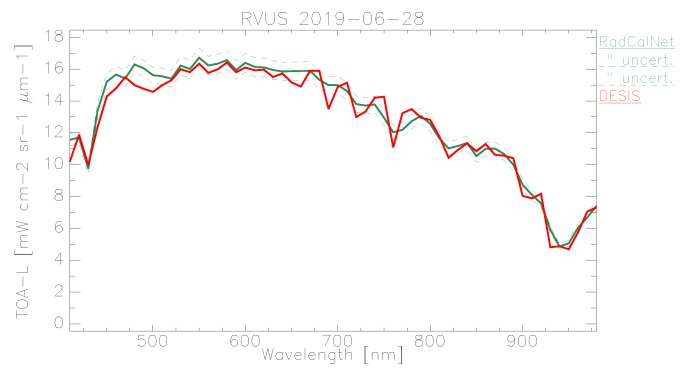
Comparison of RadCalNet TOA-L to DESIS mean TOA-L for RadCalNet location, RVUS, 28 June 2019, resampled to RadCalNet spectral resolution.

**Figure 14:**
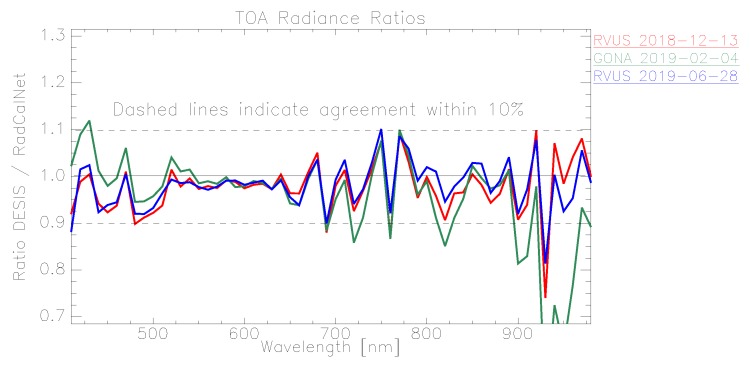
Ratio of mean DESIS TOA-L and corresponding RadCalNet data for all 3 sites, at RadCalNet spectral resolution.

In the following plots, the mean TOA radiance values from RadCalNet and DESIS are shown for the three sites and three dates (Figure 11, Figure 12 and Figure 13). In addition, the ratio $\frac{{\bar{L}}_{TOA,DESIS}}{L_{TOA,RadCalNet}}$ is shown in Figure 14.

DESIS shows good overall agreement to all three RadCalNet measurements, as it lies within 10 %except for atmospheric absorption features (Figure 14). This good agreement is even more prominent when considering the uncertainty ranges provided by RadCalNet, as depicted in Figure 11, Figure 12 and Figure 13. To put this level of agreement into perspective, the agreement among RadCalNet, Landsat-8 and Sentinel-2 A/B is reported to be within 5 % for these sites (see [113]). For the remaining differences between RadCalNet and DESIS, up to ∼525 nm, the differences are partially due to noise. From ∼525 nm to ∼650 nm, the results are excellent, which is also due to the fact that this wavelength region has a high SNR and minimal atmospheric features. Beyond these wavelengths, the results are likely contaminated by uncertainties in water vapor and atmospheric features, even more so as small wavelength calibration errors may also amplify the atmospheric differences. Finally, for wavelengths above ∼800 nm, the Etalon effect of the CMOS sensor also has an influence. In addition, discrepancies might also result from other sources including the spectral resampling steps, viewing angle and other BRDF effects, as well as slight differences within the E0 model and the actual solar irradiance. It is worth pointing out that currently the uncertainty budget for DESIS is a work in progress; when available, the radiometric uncertainty estimates will be made available at [90].

When analyzing site-specific and temporal dependencies, no clear trend can be seen. Both RVUS results are highly consistent (Figure 11 and Figure 13), while having a time difference of approximately six months. In addition, GONA results are consistent to RVUS, but show larger difference within atmospheric absorption features, especially the 950 nm feature. This might be due to inaccuracies in the RadCalNet atmospheric measurements and/or upscaling from BOA to TOA, and was communicated to RadCalNet. Since the spectral shape of the differences between DESIS and RadCalNet are consistent over approximately six months, and as the overall magnitude of these differences is also highly similar in relation to the RadCalNet uncertainty budget, it can be concluded that the DESIS radiometric calibration was stable over this period of time. In this context, it is also worth noting that GONA on 4 February 2019 and especially RVUS on 13 December 2018 have low overall radiance levels due to large SZA (Table 4), but this does not significantly affect the level of agreement.

#### 4.2.2. Top-of-Atmosphere Validation against other Missions

Since the number of coincident ground truth datasets were limited, and to have independent checks of radiometric accuracy, DESIS was also compared to other satellite sensors in addition to comparing against RadCalNet. Cross-calibration with well-calibrated sensors is a common technique used for radiometric calibration and validation. Landsat-8 Operational Land Imager (OLI) and Sentinel-2 MultiSpectral Instrument (MSI) (both Sentinel-2A and Sentinel-2B) were used to perform the cross-calibration for DESIS [114,115]. Although these satellites acquire multispectral data rather than hyperspectral data, coincident comparisons to other sensors with similar viewing geometries can be made by integrating DESIS hyperspectral bands to match bandpasses and by using scenes with slowly varying reflectance spectra. Landsat-8 data have 30-m GSD and five spectral bands within the DESIS range, while Sentinel-2 has four 10-m bands, four 20-m bands, and one 60-m band within the DESIS spectral range. This comparison was performed using the DESIS L1B 2.55 nm full resolution hyperspectral data. The USGS EarthExplorer archive was used to identify near simultaneous nadir acquisitions over pseudo-invariant sites between DESIS and Landsat-8 or Sentinel-2. Bright, highly reflective scenes were used, which minimized the impact of the atmosphere on TOA measurements. Pseudo-invariant sites were selected since they change slowly and are often approximately Lambertian. In most cases, acquisitions occurred within one hour of each other. Small sensor zenith angles (near nadir) were preferentially selected when possible to reduce atmospheric and BRDF differences between datasets. Table 5 lists the acquisitions used for comparison, including site and acquisition parameters. The Barreal Blanco, Argentina DESIS acquisition, shown in bold, had both near-coincident Landsat-8 and Sentinel-2 data on the same day.

To reduce the differences associated with solar zenith angle, comparisons were made using TOA reflectance. Sentinel-2 L1C data was provided in TOA reflectance. DESIS L1B and Landsat-8 L1TP TOA radiance were converted to reflectance using the equation:

(3) $$\rho_{TOA}=\frac{\pi \;L_{TOA}\;d^{2}}{\cos(SZA)\;E0}$$

where the variables are as described above in Section 4.2.1.

**Figure 15:**
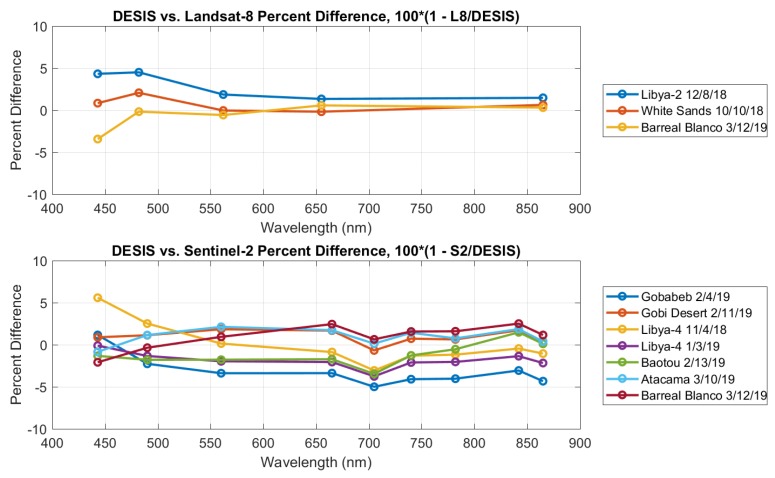
Differences (%) between DESIS and Landsat-8 (**top**) and Sentinel-2 (**bottom**) TOA reflectance for all sites.

DESIS data were band integrated to match the spectral resolution of the Landsat-8 and Sentinel-2 data. The multispectral sensor spectral responses were first interpolated to match the DESIS 2.55 nm hyperspectral wavelengths. Then, integrated reflectance was calculated for each multispectral band using the following equation:

(4) $$\rho_{band}=\frac{\int \rho \left(\lambda \right)\;RSR_{band}\left(\lambda \right)\;d\lambda }{\int RSR_{band}\left(\lambda \right)\;d\lambda }$$

where ρ(λ) is the DESIS hyperspectral TOA reflectance, $RSR_{band}\left(\lambda \right)$ is one band of a sensor spectral response interpolated to match the DESIS wavelengths, and $\rho_{band}$ is the band integrated TOA reflectance value. Comparisons were made for all images using a 210-m × 210-m reference area around the defined latitude/longitude of the pseudo-invariant sites. All reference areas were visually identified in the satellite imagery, and pixels within the area were averaged to produce a mean reflectance value per spectral band. In all but one case, the TOA reflectance difference between DESIS and the comparison sensor was less than ±5%. In the remaining case, Libya 4 on 4 November 2018, DESIS TOA reflectance was 6% different from the Sentinel-2A 443-nm band. However, this acquisition had the largest DESIS sensor zenith angle, which could impact TOA measurements, especially in the blue end of the spectral range. Figure 15 summarizes the differences from Landsat-8 and Sentinel-2 for all sites.

Several of the Landsat-8 and Sentinel-2 band center wavelengths are very similar (Coastal aerosol ∼443 nm, Blue ∼482 nm, Green ∼562 nm, Red ∼655 nm and NIR ∼865 nm). For these bands, the cross-calibration differences (%) from DESIS for both sensors were combined (Table 6). The mean differences between DESIS and the comparison sensors were all < 1%, showing very little bias in the radiometric accuracy, and, as expected, the largest variation was seen in the lower wavelengths.

#### 4.2.3. Signal-to-Noise Ratio

**Figure 16:**
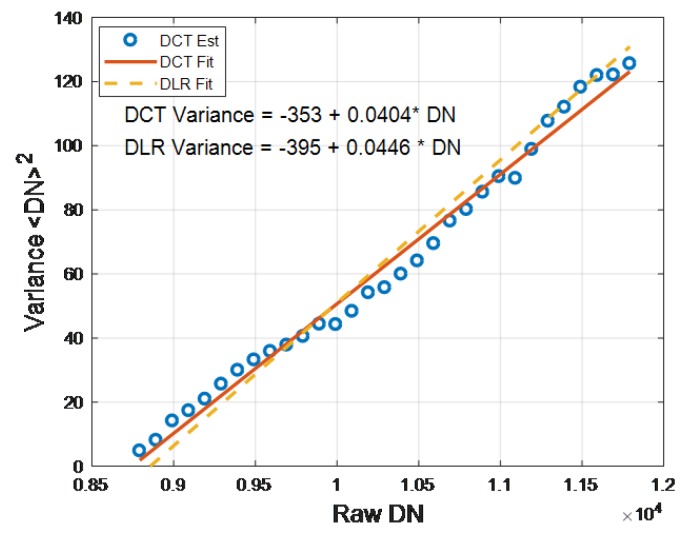
Mean–variance plot derived from a portion of the Sudan image acquired on 11 April 2019, showing agreement with ground-based measurements.

A Discrete Cosine Transform (DCT) technique was used to estimate the SNR of unbinned 2.55 nm spectral sampling DESIS L1A in-flight imagery. Photon Transfer techniques are typically used preflight to measure SNR over the entire dynamic range [116]. Directly using inflight data is usually problematic since the generally unknown scene variation must be accounted for. Using DCTs or other similar compact transforms allows the separation of low frequency scene variations from the white read and photon noise [117]. Since DCTs are compact transforms, they require only a few low order coefficients to describe scene variations [118] and the higher order DCT coefficients are more likely to be noise and can be used to relate noise in the frequency domain to noise in the time domain. L1A imagery was analyzed since it is most directly correlated to sensor read and photon noise. To avoid the fixed pattern noise within the image product, the technique performs 1-D DCTs along individual columns. A running 1-D DCT using 16-point segments is computed in the along-track columns. The means of each segment are estimated and binned and the highest order DCT coefficient (e.g., 16th) is assigned to the bin. The variance of the binned highest order DCT coefficients is then estimated after outliers identified by Chauvenet’s criterion [119] and removed. The resulting mean and variance of each bin are used to generate a mean–variance plot. Early assessments have utilized relatively uniform scenes (pseudo-invariant sites and water), and avoid any defect pixels. To improve the ability to span the full dynamic range in these early, relatively homogeneous scenes, analysis is performed in the DN space rather than in radiance. An example mean–variance plot is shown in Figure 16 for part of an image of the Sudan pseudo-invariant site taken on 11 April 2019. The resulting plot was in excellent agreement with the ground-based DESIS measurements [120]. These ground-based measurement’s mean–variance plots predict SNRs>200 for a MODTRAN calculated TOA spectral radiance with a Mid-latitude Summer (MLS) atmosphere, 30% albedo, 45 degree solar elevation, rural aerosol and 23 km visibility.

### 4.3. Spectral Calibration and Properties

Spectral characterization of the instrument is essential, especially when the measurement data are linked to the atmospheric transmission spectra, as in L2A processing. Inaccuracies of tenths of nanometers in the spectral response can lead to strong distortions in the derived reflection spectra, especially in the region of atmospheric absorption bands.

Spectral calibration of the DESIS instrument was performed in the laboratory and provided the baseline for spectral referencing of the DESIS data. Through vicarious calibration, a minor update of the spectral referencing was performed with respect to the pre-flight calibration. In addition, the analysis of the OBCA spectral calibration data shows that the spectral stability of the instrument is better than 0.2 nm (RMS) over the whole spectral range of the instrument.

Together with the spectral stability, two other effects severely influence the spectral response of DESIS and shall be corrected during data processing: the rolling shutter effect and the spectral smile effect. The next two sections present the corrections employed and their results.

#### 4.3.1. Rolling Shutter Correction

**Figure 17:**
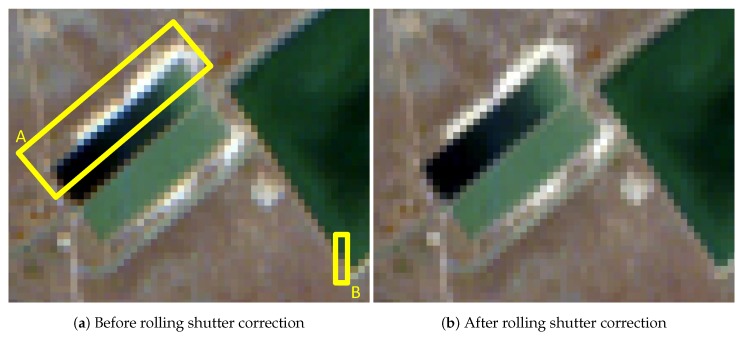
Intermediate DESIS L1B product before (**a**) and after (**b**) rolling shutter correction. Region A presents a bright blue line of pixels in the transition between different surfaces before applying the correction. The line disappears in (**b**) when the rolling shutter correction is applied. Region B encloses an area containing clear water and soil pixels, along with a bordering pixel that delimits both regions. The effect of the rolling shutter correction on the spectral profiles of Region B pixels is shown in Figure 18.

**Figure 18:**
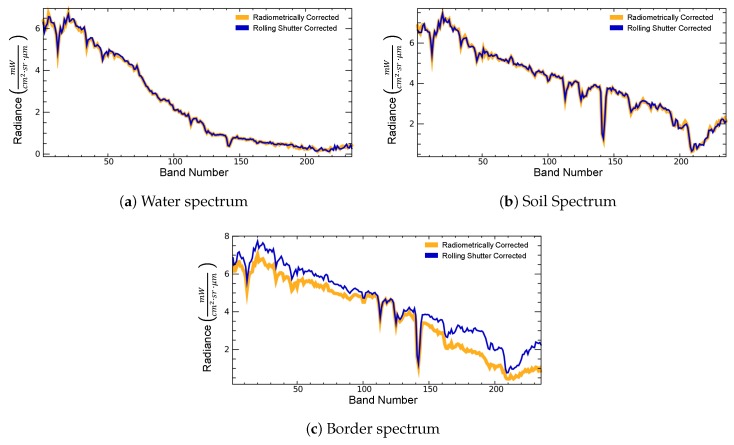
Intermediate DESIS L1B product spectra from the Region B defined in Figure 17. The effect of the correction on homogeneous pixels is negligible, as shown in (**a**,**b**). In (**c**), the effect of the rolling shutter correction is more visible, because for some bands the instrument captured a different surface.

To correct the drawbacks of the use of the rolling shutter acquisition mode, an interpolation on the along-track direction is performed (see Section 3.1). The effects of the correction at image level can be seen in Figure 17. Figure 17a shows a DESIS L1B intermediate product that has been radiometrically corrected and Figure 17b shows the same product after the rolling shutter correction. When analyzing the borders between surfaces, the post-rolling shutter corrected image presents clearer or sharper transitions. This is especially noticeable in the defined Region A, where a bright blue line of pixels appears before applying the correction. For the comparison of spectral profiles before and after correction, Region B encloses an area containing clear water and soil pixels, along with a bordering pixel that delimits both regions. Figure 18 shows the corresponding spectra before and after rolling shutter correction. In this specific case, the border pixel shows a clearer soil spectrum after the reconstruction, similar to the spectra in Figure 18b.

#### 4.3.2. Spectral Smile Correction and Validation

Taking advantage of the narrow and continuous spectral sampling of imaging spectrometers, the center wavelengths’ positions can be vicariously validated and, if required, also accurately re-calibrated based on narrow atmospheric absorption features (see, e.g., [121,122]). According to a sensitivity analysis by Guanter et al. [121], the typical accuracy of such approaches is about ±0.2 nm, and therefore useful in context of DESIS’ spectral sampling of ∼2.5 nm.

Hence, for the validation of DESIS data, the following methodology was developed. Using the MODTRAN radiative transfer code ([103]), the typical radiance spectrum in the range of the the Oxygen A feature between 741 nm and 788 nm is calculated with a 0.1 nm spectral resolution. Within this wavelength range, the influence of aerosols, water vapor and other atmospheric constituents is relatively small and can be neglected. Next, this fine resolution spectrum is spectrally resampled to the DESIS spectral resolution using the nominal bandwise FWHM information. For the assumed center wavelengths, a total of 80 center wavelength positions are used relating to shifts within ±2.0 nm around the nominal band centers, in steps of 0.05 nm. Then, the bandwise column means of L1B Earth data-takes were calculated, resulting in a “Detector Map” (DM), which represents the mean radiance that each detector element received during the data-take ([123]). For the bands close to and within the Oxygen A absorption band, the 80 normalized MODTRAN-simulated spectra are matched to the normalized DESIS spectra. The best match between the normalized radiance spectra can then be related to a center wavelength.

For the validation of the DESIS spectral calibration, data from different calibrations and processing steps are analyzed. Within Table 7, the results of this analysis are shown for the Gobabeb scene from 4 February 2019, which was found to be representative. In general, the laboratory-based spectral calibration already results in an average spectral shift of ∼0.5 nm for this wavelength range, which can be further improved when applying the spectral smile correction to ∼0.4 nm. Using the updated vicarious in-orbit calibration, these shifts are significantly reduced to ∼0.2 nm for both processing levels.

While the improvement of the post-launch vicarious calibration is clear, the effect of the smile correction procedure is needs further analysis. Assuming a perfectly smile-free system, the center wavelengths position should be identical for all cross-track pixels. In the case of spectral smile, each cross-track element senses a slightly different wavelength position, and also with a slightly different spectral response function (SRF). Thus, the atmospheric absorption feature is resolved differently for different cross-track positions and is used in a separate matching procedure for each cross-track element. In Table 8, the wavelength calibration is given for different cross-track elements across the FOV for the standard DESIS product (using the vicarious calibration and smile-correction applied). In general, the correction of spectral smile further improves the spectral calibration, but, as can be seen for the cross-track elements towards the edge of the detector, there are also bands and cross-track positions where the spectral smile correction is increasing the calibration errors. Nevertheless, these differences of standard DESIS products compared to the validation results are always within ∼0.9 nm. Keeping in mind the DESIS spectral resolution of ∼2.5 nm and the typical accuracy of this approach, this equates to a maximum error of ${}^{1}{\;/\;}_{3}$–${}^{1}{\;/\;}_{2}$ of a spectral pixel. In addition, for the standard 10 nm binning products, these spectral shifts are not relevant.

When analyzing multiple scenes, the overall consistency in the wavelength calibration of the smile-corrected products is ∼0.55 nm, calculated as the standard deviation of the wavelength differences of single detector elements compared to their nominal center wavelengths. The standard deviation over the various cross-track elements is similar, indicating that the magnitude and shape of the spectral smile are constant and can be corrected.

**Figure 19:**
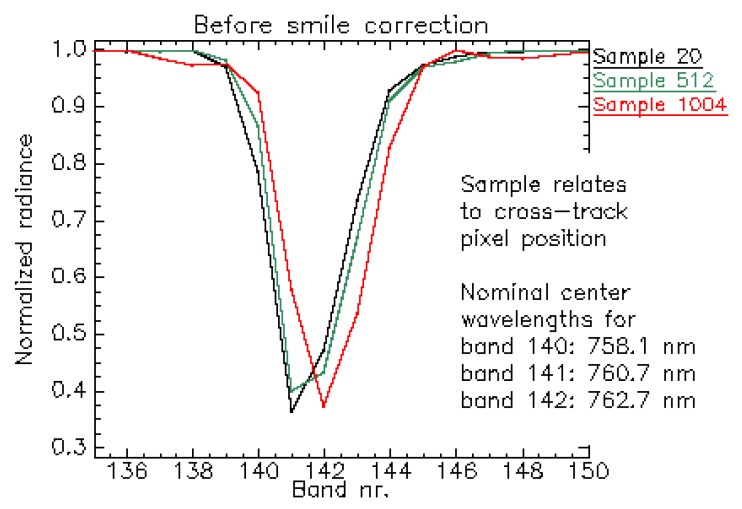
Example of the cross-track derivation of the Oxygen A absorption feature before smile correction.

**Figure 20:**
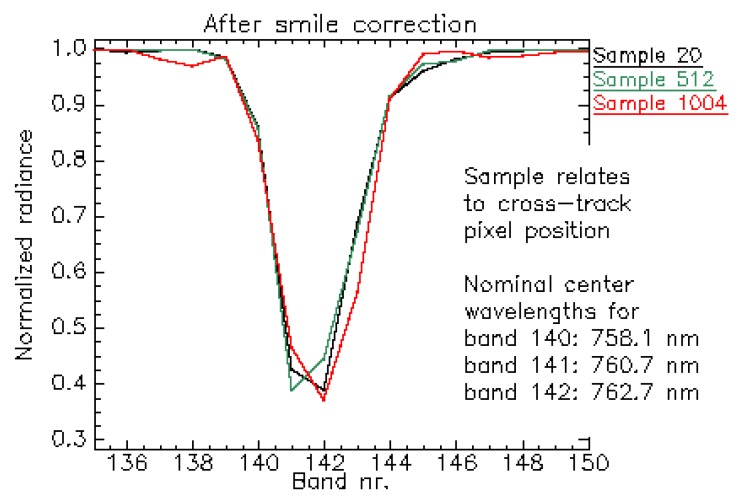
Example of the cross-track derivation of the Oxygen A absorption feature after smile correction.

For illustration, Figure 19 depicts the normalized radiance spectra over a homogeneous scene for cross-track positions 20, 512 and 1004. After applying the smile correction (Figure 20), the feature is more consistently resolved, indicating that the correction mitigates the spectral smile effect. However, as described before, for some bands and cross-track positions the smile correction is only accurate within ${}^{1}{\;/\;}_{3}$ of a spectral pixel.

### 4.4. Geometric Properties

In the following sections, the actual spatial resolution in terms of the Modular Transfer Function (MTF) is examined and compared with the laboratory measurements, and the geolocational accuracy is derived based on the evaluation of a series of images collected during the commissioning phase. Correctly georeferenced and registered images are needed for multi-sensor and multi-temporal image fusion, overlaying images on existing datasets or maps, change detection, map updating, and integration into geographic information systems.

#### 4.4.1. Modulation Transfer Function MTF

**Figure 21:**
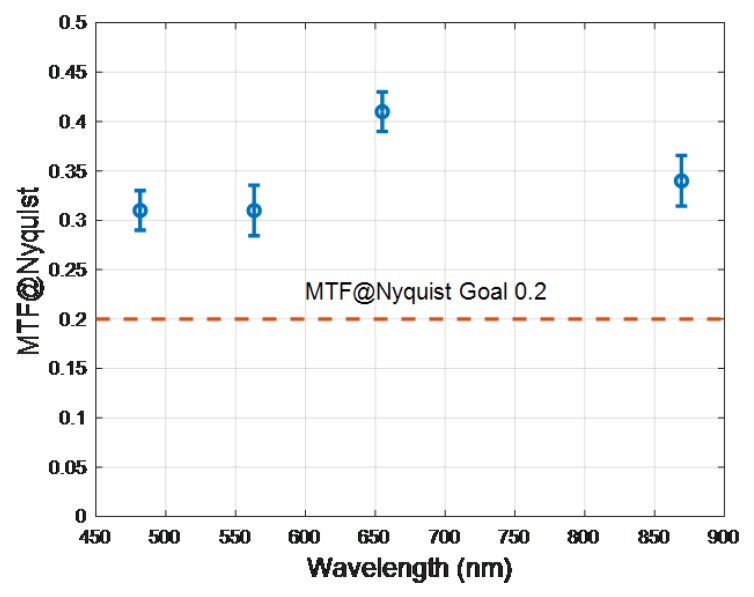
DESIS in-flight MTF@Nyquist in the cross-track direction. Values plotted are means and standard errors of 30 in-scene edges.

**Figure 22:**
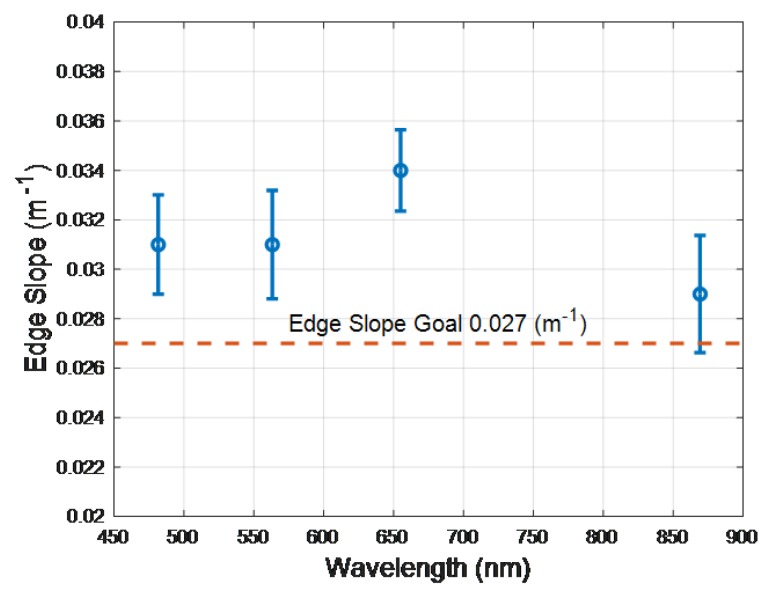
DESIS in flight Edge Slope in the cross-track direction. Values plotted are means and standard errors of 30 in-scene edges.

The spatial resolution of the DESIS Level 1B data product in terms of Modulation Transfer Function at Nyquist (MTF@Nyquist) and Edge Slope (ES) was estimated for 10.2 nm spectrally binned data in the blue, green, red and NIR bands. DESIS spatial resolution is specified in term the MTF@Nyquist with a goal to exceed 0.2. The Landsat community, on the other hand, uses Edge Slope (ES), which is defined as the average edge slope between the 40% and 60% points of a normalized edge response [124], and, as a reference, is required to exceed 0.026 m${}^{-1}$. Most bridges run north–south or east–west and are often used as pulse targets to estimate the spatial resolution for moderate resolution satellite imagers such as Landsat [125]. Because the ISS has a 53-degree inclination, cardinal oriented targets cannot be used to estimate along-track and cross-track spatial resolution parameters. Instead, DESIS cross-track spatial resolution was estimated using a set of large agricultural fields orientated in such a way as to create near ideal cross-track edge responses. A tilted edge response technique [126] was applied to these responses to determine MTF@Nyquist and ES. A benefit of this technique is that it generates a sampled edge response while minimizing aliasing. For this assessment, an automated tilted edge response algorithm was employed that: identifies potential edges within a scene where image gradients are maximized; screens identified edges for length, orientation, and uniformity; fits resulting data using a functional form which is the sum of an error function and fifth order polynomial; and then calculates MTF@Nyquist and ES. In this functional form, the error function portion of the fit models the majority of the edge response variation and the polynomial portion fits the small deviations from an ideal error function. Example mean results for 30 found edges are shown in Figure 21 and Figure 22. The cross track MTF@Nyquist exceeds 0.3 and the DESIS goal of 0.2 and is higher than the minimum ES set for Landsat systems. Future work will evaluate spatial resolution in the along-track direction, which we expect will be slightly lower than the cross-track direction.

#### 4.4.2. Geolocation Accuracy

As mentioned in Section 3.2, GCPs are generated for each DESIS scene using image matching employing reference images in order to improve the parameters of the geometric sensor model. If this on-the-fly method fails, parameters derived from statistical evaluation of a series of previous acquisitions are used. In this case, thermal influences on the MUSES/DESIS system and uncertainties in the attitude and position measurements result in worse geometric accuracy. For the assessment of the geolocation accuracy and boresight angle determination, 177 scenes were analyzed. These scenes were acquired globally with off-nadir viewing angles up to $26^{\circ }$ and solar zenith angles up to $73^{\circ }$. On average, 210 GCP per scene were found for the improvement of the sensor model parameters and 968 CP per scene for accuracy assessment. The achieved linear Root Mean Square Error with respect to the reference scenes based on the evaluation of individual scenes is given in Table 9 and is below one pixel size (0.7 pixel).

**Figure 23:**
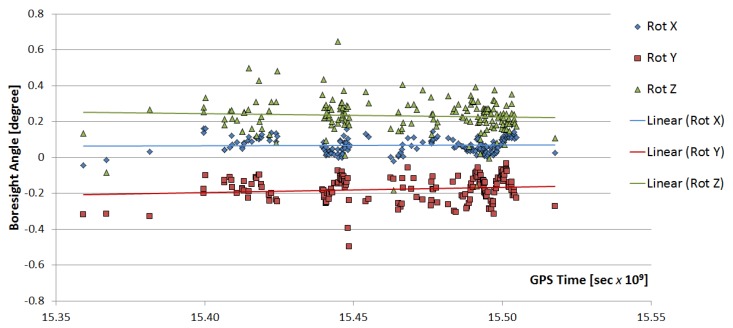
Estimated boresight angles of the DESIS sensor with respect to the body coordinate frame. The three angles as well as the trend lines are plotted against the acquisition time of the data.

The assessment of absolute geolocation accuracy requires high quality GCPs distributed throughout the world and scenes acquired under different conditions. Such an investigation still needs to be carried out, but considering the geolocation accuracy of the reference data (see Section 3.2), an upper limit of the absolute linear RMSE value of about 28 m can be estimated. Relying only on the calibrated sensor parameters and on-board measurements, the geolocation precision is about 298 m across track and about 496 m along track. The differences in precision are probably due to the fact that along track the rotating mirror (POI) of DESIS shows a certain inaccuracy. Figure 23 demonstrates the stability of the estimated boresight angles over a time range from 11 September 2018 to 5 March 2019. The three rotation angles (X, around along track; Y, around across track; and Z, around optical axis) from the sensor coordinate frame to the body frame show no trends during this six-month period with the mean values ($rot_{x}^{mean}$, $rot_{y}^{mean}$, $rot_{z}^{mean}$) = ($0.0692^{\circ }$, $-0.1753^{\circ }$, $0.2323^{\circ }$) reflecting the boresight calibration and the standard deviation ($rot_{x}^{stdv}$, $rot_{y}^{stdv}$, $rot_{z}^{stdv}$) = ($0.0427^{\circ }$, $0.0711^{\circ }$, $0.0981^{\circ }$) reflecting the uncertainty if no matching can be performed.

### 4.5. Surface Reflectance and Atmospheric Properties

The accuracy of the BOA surface reflectance depends on the accuracy of the instrument calibration, the input parameters (such as radiative transfer functions, ozone column, and DEM) and the determination, during the atmospheric correction process, of the atmosphere parameters (mainly WV and AOT).

In the next sections, we present strategy, datasets and results for surface reflectance validation. In a first step, the atmospheric parameters calculated by the processor, and later used for the surface reflectance determination, are validated against measurements of the atmosphere from ground. Subsequently, BOA surface reflectance values are validated against in situ measurements.

#### 4.5.1. Aerosol Optical Thickness and Water Vapor

The validation of these products is done using the AErosol RObotic NETwork (AERONET) global dataset of sun-photometers [127], level 1.5. It is performed according to several overpass (atmosphere-based) criteria that foresee the same atmospheric conditions in both datasets:

- Space: The DESIS measurement is extracted from a 9 km × 9 km region of interest (here after called ROI${}_{9}$) around the AERONET coordinates.
- Time: The AERONET measurement is interpolated to the overpass time of DESIS through the AERONET station coordinates.
- Wavelength: The AERONET AOT data are interpolated to 550 nm wavelength.

Once the dataset fulfills the overpass criteria, the AOT and WV (calculated per pixel) are calculated using the following pixel selection dependent on atmospheric parameters:

- AOT is extracted from the DDV pixels detected in the scene.
- WV is extracted from clear land pixels.

Scenes having fewer than 5% of the pixels inside the ROI${}_{9}$ around the AERONET station fulfilling the mentioned criteria for all parameters were not included in this study.

From all the scenes fulfilling the atmosphere-based overpass conditions, 11 contain more than 5% DDV pixels and 36 have more than 5% clear land pixels in the ROI${}_{9}$. Those has been used for the validation study of the AOT and WV, respectively. Calculating the RMSE of the absolute difference of the AOT and WV with respect to AERONET, we get an RMSE for the aerosol optical thickness (AOT) of 0.17% and 11% for the water vapor column (WV).

The validation results of the WV retrieval algorithm, using the water absorption region of 820 nm, are consistent with the APDA algorithm validation performed by Richter and Schlapfer [128], who estimated an RMSE of 10% for Sentinel-2 data, using the 940 nm bands.

The validation results for the AOT show a RMSE larger than the typical one of 0.08 reported by Obregón et al. [129] when comparing Sentinel-2 with AERONET stations. One possibility could be a difference of the spectral response functions used for the AOT retrieval (red and NIR). Research on Sentinel-2 data by Obregón et al. [129] showed a decrease of the AOT RMSE compared to AERONET values from 0.14 to 0.08, after an update of the spectral response functions in the blue.

#### 4.5.2. Bottom-of-Atmosphere Reflectance

To validate the Bottom-Of-Atmosphere (BOA) reflectance, we selected spectra from RadcalNet sites, on three different RadcalNet sites: La Crau (France), Railroad Valley Playa (USA), and Gobabeb (Namibia). Baotou RadcalNet site was removed from this study due to a limited site extension (<48 m). For an accurate comparison, the data-takes of DESIS and any other measurement should fulfill the following “overpass” criteria:

1. Time: Spectra comparison is only performed between data takes acquired within the same day. For RadcalNet, the TOA spectra are the interpolation result between two measurements spaced <30 min.
2. Space: Different extensions around RadCalNet measurements points are considered depending on the site surface reflectance homogeneity specifications (see Table 10). The extension considered in this study is specified in a separate column in the table.
3. Wavelength: The spectra of all the sensors are convolved to the SRF of the sensor per band with larger FWHM (in this case, RadcalNet) (see Equation (5)).
  (5) $$\rho_{DESIS,RCN}=\frac{\int_{RCN_{\lambda }}\rho_{DESIS}\;\cdot \;SRF_{RCN,\lambda }}{\int_{RCN_{\lambda }}SRF_{RCN,\lambda }}$$

In addition to the overpass criteria listed above, we included in this study those DESIS scenes acquired under good atmospheric conditions. Scenes with clouds and visible cirrus were excluded from the validation dataset. This is the case for the acquired DESIS scenes over La Crau RadcalNet site. Therefore, only scenes acquired over two RadcalNet sites were evaluated (see Table 10). These two sites are typically arid, without any presence of dark dense vegetation. For these scenes, the atmospheric correction algorithms cannot estimate an accurate value of the AOT and a default value corrected by the DEM is used in such scenes. To validate the rest of the L2A processing, with the exception of the influence of the AOT retrieval accuracy, we used the measured AOT value at the RadcalNet site as an input to the atmospheric correction of DESIS data for validation purposes only. Note that this possibility is not available for the standard processing of L2A DESIS products.

The corresponding ozone column for each of the DESIS scenes was extracted off-line from the MODIS database MOD08_E3 (version 61) [133] and included in this validation. This option is available to all users.

Note that the DESIS BOA reflectance compared in this study does not include the accounting of Bidirectional Reflectance Distribution function at the surface or RadcalNet data.

**Figure 24:**
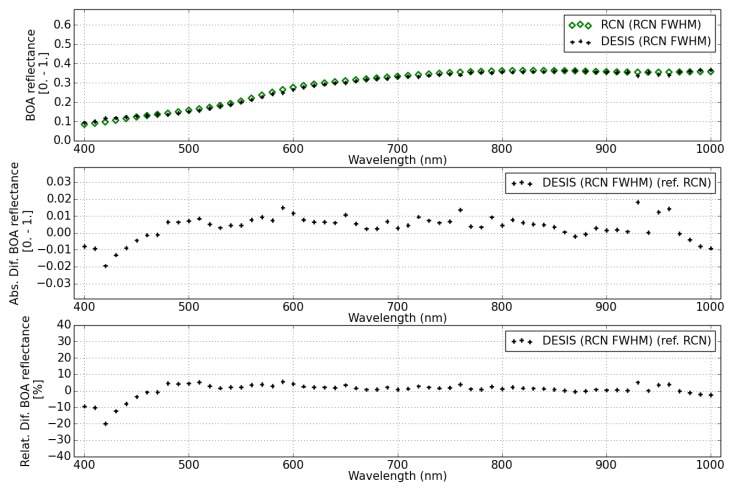
DESIS (black crosses) and RadCalNet (green diamonds) BOA surface reflectances at Gobabeb site (4 February 2019). DESIS spectrum at 2.5 nm has been convolved with RadCalNet spectral response: (**top**) BOA surface reflectance; (**center**) absolute difference of surface reflectance; and (**bottom**) Relative difference (%) of surface reflectance.

**Figure 25:**
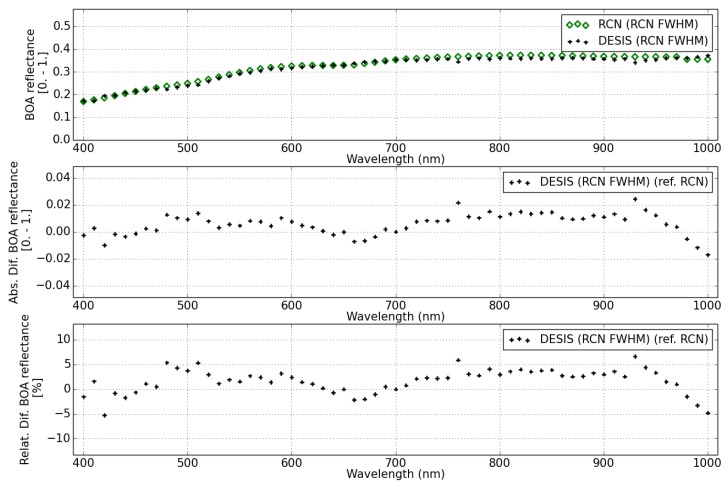
DESIS (black crosses) and RadCalNet (green diamonds) BOA surface reflectances at 10 nm spectral resolution for Railroad Valley Playa site (13 December 2018): (**top**) BOA surface reflectance; (**center**) absolute difference of surface reflectance; and (**bottom**) Relative difference (%) of surface reflectance.

**Figure 26:**
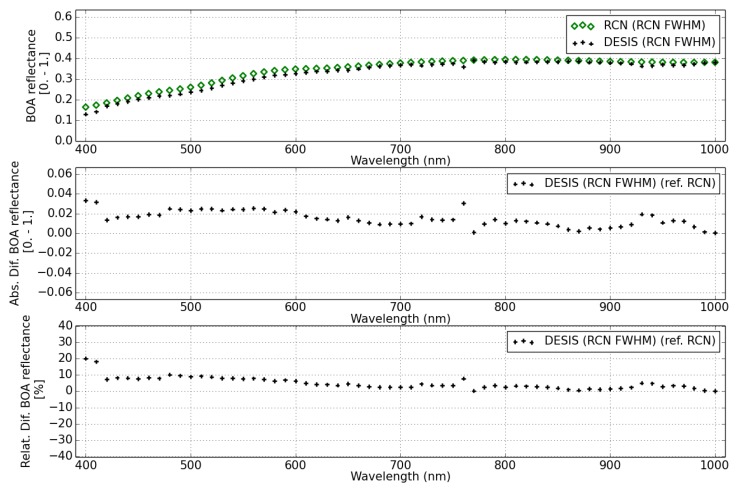
DESIS (black crosses) and RadCalNet (green diamonds) BOA surface reflectances at 10 nm spectral resolution for Railroad Valley Playa site (28 June 2019). For each of the set of plots: (**top**) BOA surface reflectance; (**center**) absolute difference of surface reflectance; and (**bottom**) Relative difference (%) of surface reflectance.

The BOA surface reflectance from DESIS and RadcalNet site Gobabeb and Railroad Valley are shown in Figure 24, Figure 25 and Figure 26, respectively.

The DESIS BOA surface reflectance is consistent with RadCalNet measurements with 10% relative difference for two different sites and for all wavelengths, except for the first bands (<420 nm) that show a difference up to 20%. This relative difference agrees with the RadcalNet site variability (see Table 10), since surface reflectance difference is calculated per pixel subtracting the RadCalNet reference spectrum for the full site extension.

**Figure 27:**
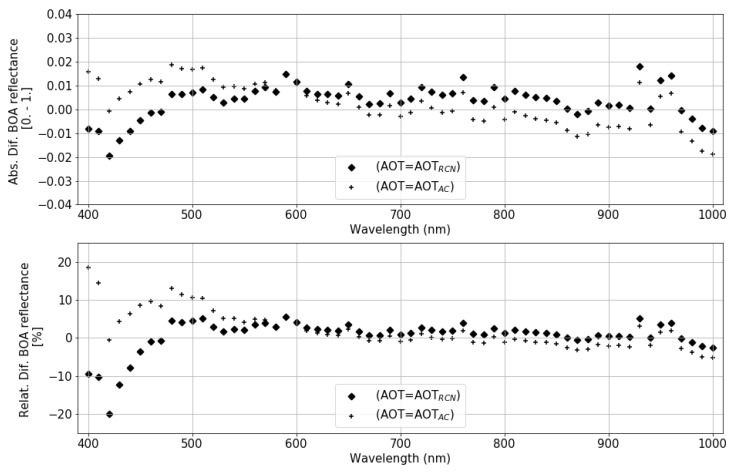
DESIS absolute (**top**) and relative (**bottom**) difference in BOA surface reflectance with RadCalNet reference data at Gobabeb site (4 February 2019).

For this type of scenes (arid sites), no DDV pixels are available to determine the aerosol content, so the atmospheric correction is performed with an estimated value. This value is rather pessimistic for RadcalNet sites, where very low aerosol content is present for those days flagged as good quality data (green dots). Figure 27 shows the order of magnitude in the estimated BOA surface reflectance for Gobabeb scene for two different values of the AOT. For this scene, the AOT measured in RadcalNet site is AOT${}_{RCN}$ = 0.06 (filled diamonds), while the value estimated for a default visibility of 23 km is of ∼0.3 (crosses). The larger difference (<25%) in surface reflectance happens in the blue wavelengths range (<480 nm), decreasing to <10% for green wavelengths. Less than a 3% relative difference is expected at NIR wavelengths.

## 5. Product Limitations

In the following section, we describe the few limitations of DESIS data which were detected during the commissioning phase, and thus reflect the current data quality. Some of these issues may be solved or mitigated in the future. Information for the improvement of the data products will be published on the DLR web portal [90].

### 5.1. Image Artifacts and Dead Pixels

Within the DESIS ground segment, the assessment of defective detector elements is conducted twice. Firstly, an interactive offline analysis is carried out as part of routine data quality control; furthermore, an online assessment of every Earth data-take is conducted by the L1B processor, and results are included in the metadata of every product. The results of the interactive analysis carried out during the DESIS commissioning phase are detailed below.

Of the 240,640 active detector elements of the DESIS CMOS detector, only minor parts cannot be used due to manufacturing defects, occurring in the first seven spectral bands on the edges of the field-of-view, as listed in Table 11. Due to the nature of these defects, no recovery of a meaningful measured signal (e.g., by extrapolation) is possible; thus, these pixels should no be used during subsequent analysis.

Whenever a single detector element consistently generates a too low or a too high signal in relation to its spatial neighboring pixels, the corresponding column in the image tile will appear darker or brighter than the neighboring columns, i.e., the image is “striped” (see Section 3). To detect and mask these detector elements surviving the de-striping process, procedures within the processing chain are used. Within the L1B processor, the bandwise column means are calculated using the uncalibrated L1A and the fully calibrated L1B data, and stored as “Detector Maps”. These DMs are arrays representing the detector chip, having the dimension of cross-track-elements × spectral bands.

The analysis was carried out using DMs from 1090 L1A and 728 L1B Earth data-takes. For the calibrated L1B datasets, no corrections for rolling shutter and spectral smile were applied, as these processing steps incorporate information from spatial and/or spectral neighboring pixels, thus partially masking the striping. Neglecting the mentioned bands having manufacturing defects (i.e., using band 8 onward), an additional 17 pixels are showing permanent defects (10 more currently under investigation), and are thus marked as “defective” and included in the dead pixel mask. All other defects which were detected this way, or were identified during the vicarious calibration, could be improved within the standard destriping procedure in the L1B processor (see Section 3.1).

Other typical image artefacts such as saturated pixels, cross-talk and missing data are rare and currently not considered as a limitation.

### 5.2. Geolocation Accuracy

As mentioned in Section 4.4.2, the geometric accuracy of L1C and L2A products can be about 15 pixels with peak values of up to 30 pixels (1 km), if refinement of the sensor model parameters by GCPs is not possible. Image matching will fail if the DESIS scene and the reference data are too different (e.g., due to a large distance in acquisition time) or do not contain sufficient texture information (e.g., rainforest areas, water areas, and snow covered areas). An increase in the instances of successful image matching could be achieved by simultaneously considering multiple DESIS scenes within a data take for image matching [134], rather than single scenes, and using a temporally up-to-date reference database, which could be retrieved from Sentinel-2 archives. Both concepts are currently under consideration.

### 5.3. On-Board Radiometric Calibration

The intended use of the on-board calibrations consisting of LEDs for future updates of the radiometric calibration tables will not be possible due to non-uniform illumination of the focal plane array [120]. Therefore, future radiometric re-calibration will only be based on vicarious calibration and cross-calibration activities.

### 5.4. Spectral Properties

As shown in Section 4.3.2, DESIS has an overall accurate and stable spectral calibration, but indications for minor wavelengths shifts and uncorrected spectral smile remain. In addition, clocking and ethaloning effects influence the bands above ∼800 nm (Section 3.1). Only for unbinned 2.5 nm resolution data, these shortcomings might affect the data analysis. Therefore, advanced correction techniques are under investigation. For binned 10 nm data, the aforementioned spectral distortions are not relevant.

### 5.5. Radiometric Properties

Shortcomings in relative radiometric calibration (i.e., striping) were successfully mitigated with the implemented striping correction (see Section 3.1). The absolute radiometric properties were successfully validated at spectral resolutions of 10 nm and coarser (see Section 4.2.1 and Section 4.2.2). At the mentioned spectral resolutions, the radiometric properties above ∼650 nm are within ∼ 10%, but could possibly be improved by an ethaloning correction and additional in-orbit calibrations. Regarding the absolute radiometric properties of DESIS (Section 4.2.1), the validation for the full spectral resolution of 2.5 nm is currently limited by the availability of suitable reference data, as RadCalNet is only providing data with 10 nm spectral resolution.

### 5.6. Masks

There are some limitations in the following L2A masks:

- Snow and clouds: Mis-classifications may result in snow pixels classified as clouds, due to a rather similar spectra both classification types for the available wavelengths of the sensor.
- The clear land mask will contain all pixels not identified by any of the different masks algorithms contained in the pre-classification routine. This is the case for DESIS concerning cirrus clouds. Some clear land pixels might contain thin and medium cirrus, as DESIS does not have any band at ∼1.38 μm. Only thick cirrus are likely to be included in the clouds mask (see lower left corner in Figure 4).
- The clouds over water mask will always be empty by default. Its values can only be filled when an external water mask is provided. Future software release could include such external water mask as input.
- Both haze masks might contain very bright water pixels.

For the best performance in the shadows determination, the usage of a DEM is required, in order to prevent some topographic shadows from not being fully recognized.

Future development efforts will concentrate on solving these issues.

### 5.7. Rugged Terrain Atmospheric Correction with Noisy DEM

It has been observed that noisy DEM regions can introduce artifacts (e.g., shadows and BRDF) into the final BOA surface reflection products. This is especially observed at very high zenith angles. Currently, there is no strategy to address this problem, therefore it is recommended to order L2A products without terrain correction for the analysis of known flat areas in combination with large sun zenith angles.

## 6. Data Fusion Experiment—An Outlook

Applications requiring high spatial resolution (e.g., for urban areas with fine structured information) will have limited usability for spaceborne imaging spectrometers with a GSD of about 30 m. This problem is typically addressed by resolution enhancement methods, which aim at increasing the spatial resolution of imaging spectrometers while preserving valuable spectral information. One option for resolution enhancement is fusing information from such images with a higher spatial resolution image containing fewer spectral bands. Resolution enhancement of imaging spectroscopy data using a multispectral image has recently gained more attention from researchers [135,136], driven by the large amount of planned satellite spectrometer missions, as well as by the rising number of applications for this kind of data. As soon as additional sensors are placed on the MUSES platform, multi-modal observations will become possible, enabling sensor fusion approaches employing the same sun–target–sensor geometry, atmospheric conditions, and object properties. In the following section, we demonstrate how the fusion of DESIS data with Sentinel-2 data leads to added value products.

The limited spatial resolution of the DESIS image data (30 m GSD) leads to single image elements that usually contain different targets on the ground. The resulting associated spectrum is then a mixture of different spectra related to pure materials (also known as endmembers); this can be a major hindrance for practical applications such as target detection, classification, and change detection, where often the desired level of detail is finer than the sensor’s GSD.

**Figure 28:**
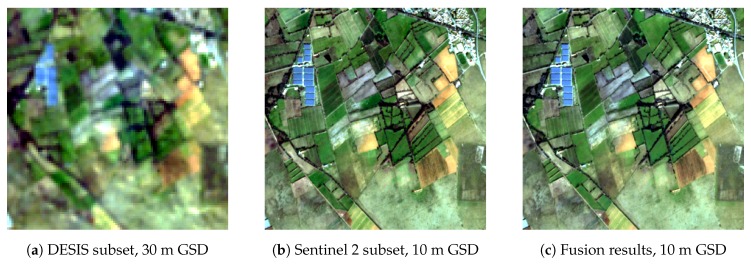
Data fusion results: true color combinations.

To produce fused data with both high spatial and spectral resolution, DESIS information could be enhanced using Sentinel-2 data, which features 12 spectral bands, four of which have a GSD of 10 m. This results in a resolution ratio of 1:3 with respect to the hyperspectral product, limiting spectral distortions in the fused products [137]. Furthermore, Sentinel-2 products are freely available through the Copernicus program, and are characterized by a high temporal resolution, allowing the retrieval of multimodal images acquired on the same area within only a few days. Both DESIS and Sentinel-2 products (only bands at 10 m GSD) were converted beforehand to BOA reflectance and accurately co-registered. As a preliminary result, an example is reported in Figure 28 for the fusion of a DESIS and corresponding Sentinel-2 image subsets acquired in the area of La Crau (France), of size 100×100 and 300×300 pixels, respectively. The fusion algorithm of choice is Coupled Non-negative Matrix Factorization (CNMF) unmixing. Both hyperspectral and multispectral data are decomposed assuming that each spectrum can be expressed as a linear combination of the endmembers present in the scene, yielding their relative fractional coverages (abundances) pixel-wise.

**Figure 29:**
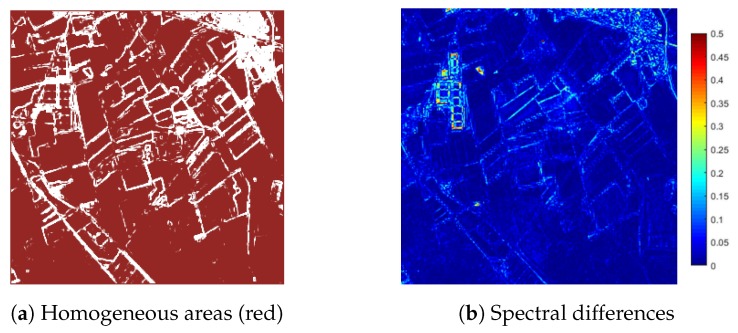
Image elements with low variance in the subset of interest, assumed to belong to homogeneous areas, reported in red (**a**). Spectral Angle between DESIS image upsampled to 10 m (nearest neighbor) and fused product (**b**). Areas with associated higher distortion usually contain mixed pixels, which exhibit different spectral features at different resolutions.

As a high resolution hyperspectral dataset on the site of interest is not available, a quantitative assessment of the fusion results is not easy. Both DESIS and Sentinel2 images can be simulated from a high resolution hyperspectral dataset; nevertheless, this would oversimplify the problem by ignoring coregistration errors, variations in atmospheric conditions, and distortions related to a single sensors [138]. To quantify the spectral distortion of the fused product, we compare them to the original DESIS data in homogeneous areas, reported in Figure 29a. These are approximately located by thresholding the variance in a local sliding window of size 3×3 in the red band of the multispectral image, corresponding to the size of a DESIS image element. Pixels with high variance are considered to be heavily mixed in the low resolution hyperspectral data and are not considered in the analysis.

**Figure 30:**
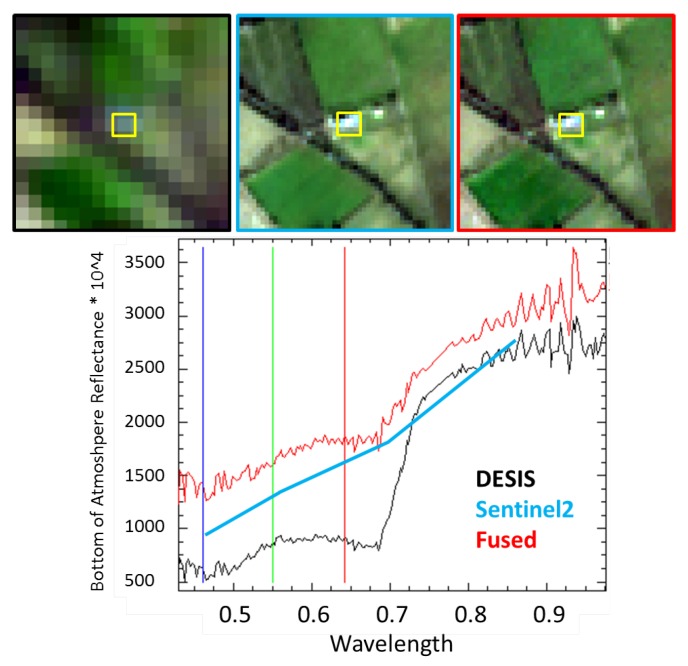
Subsets from DESIS (30 m), Sentinel-2 (10 m), and the fused product (10 m). Below are reported the relative spectra for the center of the yellow squares in the image subsets. The Sentinel-2 spectrum, corresponding to a man-made object, shows a high reflectance in the red spectral range: this is not observable in the larger DESIS pixel, dominated by vegetation. The fused product (10 m) correctly synthesizes a full spectrum resembling the general behavior of the Sentinel-2 product.

Distortion is quantified through Spectral Angle (SA) [139] and reported in Figure 29b. Pixels in homogeneous areas with associated low variance have low to no spectral distortion: the mean SA is 0.035 with a standard deviation of 0.027, and 97.5% of the pixels have a SA below 0.1, i.e., below typical thresholds for practical material detection applications. On the other hand, spectra within mixed image elements are different from their average spectrum in the original DESIS image. Such differences are usually meaningful; for example, the spectral reconstruction for a pixel exhibiting high spectral distortion is reported in Figure 30. At a spatial resolution of 30 m, the building in the image is mixed with the surrounding vegetation, resulting in a spectrum which closely resembles the latter. The multispectral pixel, on the other hand, resembles the typical ramp characterizing the spectra of man-made structures, and this information is injected into the fusion process, resulting in a spectrum exhibiting features typical of impervious surfaces.

### Target Detection

**Figure 31:**
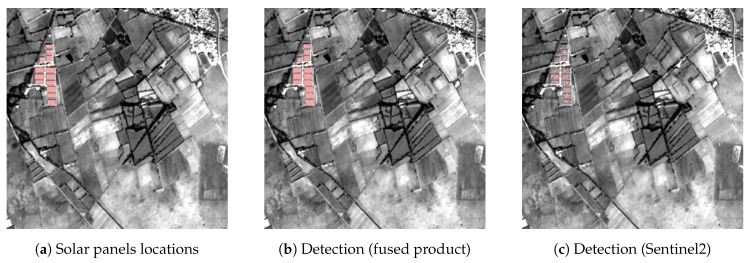
Detection of solar panels by hard thresholding of spectral similarity of each image element in the enhanced product (output of data fusion) and original multispectral dataset.

To quantify the enhanced spectral characterization of each image element, we report in Figure 31 a target detection application. A sample spectrum is selected from the same location for the solar panels to the NW of the image subset for the hyperspectral and multispectral images. Subsequently, pixels with a SA distance smaller than 0.02 rad are selected as belonging to the solar panels class. The results show that Sentinel 2 has problems in detecting the class of interest solely based on spectral similarity with such a low threshold, while this is not the case for the fused product. Quantitative results in terms of overall accuracy and percentage of false alarms are reported in Table 12. Even though in the fused products the false positives increase by a factor of 10, these are mostly located on the borders of the panels which were not included in the ground truth.

When applying data fusion algorithms as in the previous examples, the following aspects must be taken into account. First, the coregistration between multispectral and hyperspectral images should be as precise as possible. In the reported example, this has been manually refined from the georeferenced products. Furthermore, noisy bands should be removed from the hyperspectral image before the fusion process, in order to prevent the unmixing-based algorithm from selecting spurious endmembers: these have been identified as bands [1–11,137–148,226–235] for the DESIS scene above; in general, special care should be taken for any application involving spectral unmixing which uses the mentioned bands. Finally, better results are to be expected when restricting the fusion to a DESIS image subset rather than a full scene: keeping the complexity of the image low, in terms of the number of materials contained, helps minimizing the spectral distortions in the fused product.

## 7. Conclusions

In May 2014, the German Aerospace Center (DLR) and the US company Teledyne Brown Engineering, Inc. (TBE) agreed to install and operate the imaging spectrometer DESIS on board of the International Space Station (ISS). The instrument, built by DLR, is the first of four possible camera systems that can be hosted by the MUSES platform. In August 2018, the DESIS spectrometer was integrated into MUSES, which marked the start of the commissioning phase. The DESIS on-orbit functional tests were successfully passed and the DLR-built processing systems installed at DLR and TBE are stably producing L1B, L1C and L2A products. Now, about five years since mission kick-off, the operational phase has been entered and the distribution of the data to the scientific and commercial community has begun. In this article, the main outcomes of the commissioning phase are presented, which are summarized as follows:

- Absolute radiometric calibration is well within 10% at the TOA radiance and TOA reflectance level when validated against RadCalNet, Sentinel-2 and Landsat-8.
- Spectral calibration after smile correction is typically better than 0.5 nm, and always within 1/3 of a spectral pixel.
- SNR is greater than 200 in the green spectral region for a 30% albedo, 45 degree solar elevation, MLS 23 km visibility, and rural aerosol TOA radiance.
- MTF@Nyquist (across track) is about 0.3–0.4.
- Geometric accuracy with respect to reference is 20 m (<1 pixel) linear RMSE in the case that GCPs can be derived from image-to-image matching; otherwise, RMSE is 300–500 m.
- BOA reflectance is within <10% based on RadCalNet, Pinnacles, and Sentinel-2 comparisons.
- The current limitations in the use of the various DESIS data products herein are described, as well as future work and improvements.

Despite the discussed limitations of the instrument, DESIS has several advantages compared to other imaging spectrometers. The high spectral sampling of 2.55 nm with a SNR of about 200 is currently unique and allows investigations of very narrow band features in the spectrum reflected from a target on ground. The imaging spectrometer DESIS will enable new applications in addition to the ones described in Section 2. The off-nadir capability of DESIS with $\pm 15^{\circ }$ along the track during a data-take enables investigations of multiangular characteristics of objects on Earth, thus providing additional target-specific information at high spectral resolution. Furthermore, the variable recording times due to the non-polar or non-sun-synchronous orbit allow investigations, e.g., of dynamics of solar-induced chlorophyll fluorescence and photosynthesis, which is subject to strong daily fluctuations. Finally, as soon as additional sensors are placed on the MUSES platform, multi-modal observations will become possible, enabling cross-calibration and sensor fusion approaches employing the same sun-target-sensor geometry, atmospheric conditions and object properties.

## Figures and Tables

**Figure 8:**
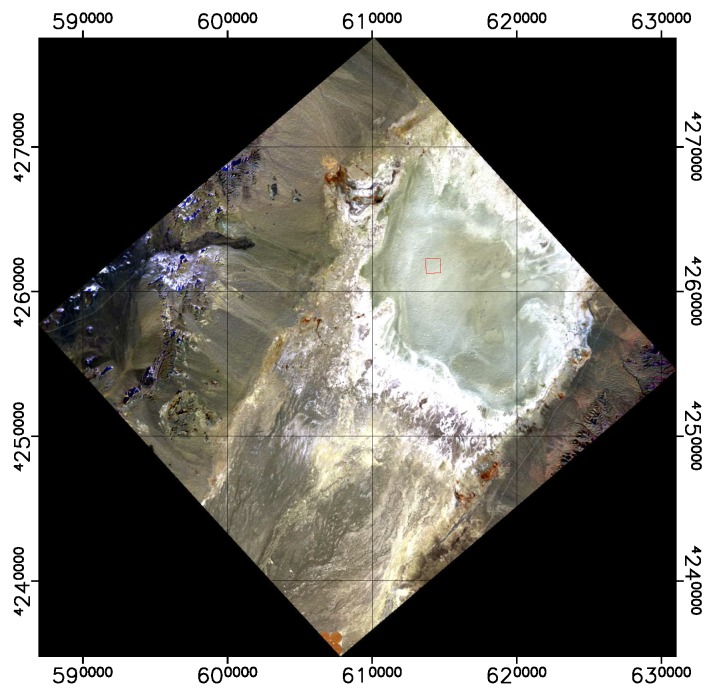
RVUS, 13 December 2018, L2A CIR image, non-linear stretch; coordinates given in WGS-84, UTM Zone 11 N. RadCalNet location as red polygon.

**Figure 9:**
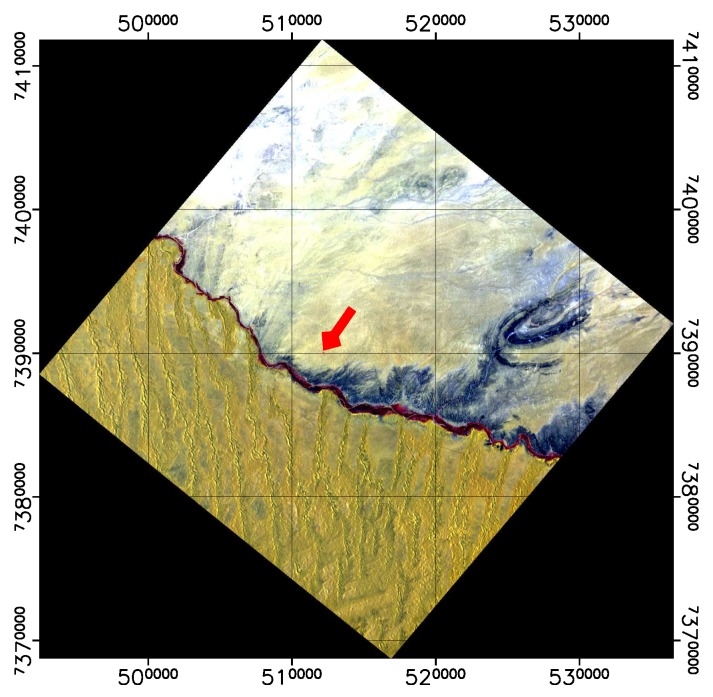
GONA, 4 February 2019, L2A CIR image, non-linear stretch; coordinates given in WGS-84, UTM Zone 33 S. Arrow pointing towards the RadCalNet location (red dot).

**Figure 10:**
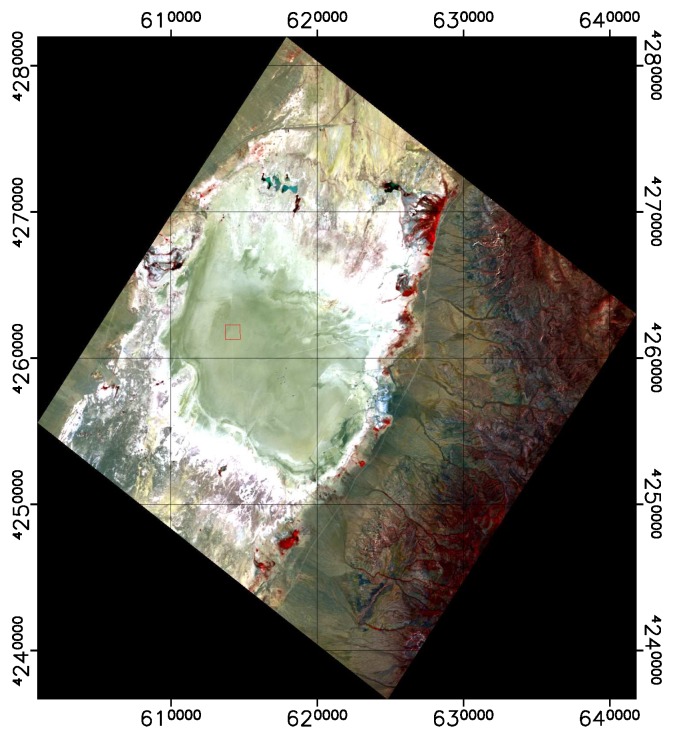
RVUS, 28 June 2019, L2A CIR image, non-linear stretch; coordinates given in WGS-84, UTM Zone 11 N. RadCalNet location as red polygon.

**Table 1:** DESIS products and user selectable processing parameters.

Product Type	Description	Order Parameters
L1B	Radiometric and sensor specific corrected data	Spectral Binning
	Top of Atmosphere (TOA) radiance (mW·cm${}^{-2}$·sr${}^{-1}$·μm${}^{-1}$)	
	All metadata attached for further processing	
L1C	L1B data orthorectified and resampled to a specified grid	Map Projection Resampling
	using global SRTM 1 arcsec DEM for terrain correction	
	using global Landsat ETM+ references for sensor model refinement	
L2A	L1C data atmospherically corrected (reflectance)	Terrain Correction Ozone Column
	using global SRTM 1 arcsec DEM for topographic correction	
	generating several masks (water, land, cloud, shadow, …)	

**Table 2:** Description of the possible abnormal pixel types.

Defect	Description
Unreliable Calibration	Focal plane element whose characterization is flagged as unreliable during on-board calibration.
Manufacturing Defects	Focal Plane element whose characterization on-ground was not nominal.
No Data	Pixel containing no information. This type of abnormal pixel only appears on initial and final tiles of a data-take due to the lack of data when L1A processor produces the tile overlap.
Low Radiance	Pixel flagged by the quality monitoring due to an abnormal low radiance value.
High Radiance	Pixel flagged by the quality monitoring due to abnormal high radiance.
Suspicious Pixel	Pixel flagged by the on-board calibration processor as not nominal but its response is yet under investigation.
Dead	Pixel which produces no response.

**Table 3:** Quality mask layers of the L2A product.

Layer	Description
1	Shadows: mask flagging those pixels identified as cloud and topographic shadows.
	The latest is not included if the user process the image without TerrainCorrection option.
2	Clear land: pixels flagged in this mask have not been identified neither as water, nor as cloud pixels.
3	Snow
4	Haze over land
5	Haze over water
6	Cloud over land: this actually contains all cloud pixels
7	Cloud over water: this layer is only filled when an external water mask is provided
8	Clear water: water detected pixels not flagged as haze
9	Aerosol Optical Thickness (at 550 nm)
10	Water Vapor (in cm)

**Table 4:** Example of DESIS scenes of RadCalNet sites used for TOA-L validation within this study. SZA, solar zenith angle; VZA, sensor view zenith angle; RVUS, Railroad Valley, USA; GONA, Gobabeb, Namibia.

DESIS Scene	Date	SZA	VZA	L2A Quicklook	RadCalNet Output Version
RVUS, Tile 2	13 December 2018	64.0${}^{\circ }$	0.8${}^{\circ }$	Figure 8	2.04
GONA, Tile 2	4 February 2019	35.3${}^{\circ }$	3.9${}^{\circ }$	Figure 9	2.02
RVUS, Tile 3	28 June 2019	19.0${}^{\circ }$	3.4${}^{\circ }$	Figure 10	2.04

**Table 5:** Near-coincident DESIS and Landsat-8 or Sentinel-2 acquisition information.

Site	Date	Lat/Lon (${}^{\circ }$)	Sensor	Time	Zenith Angle (${}^{\circ }$)			
				Difference	Sensor		Solar	
				(min)	DESIS	L8/S2	DESIS	L8/S2
Libya 2	8 December 2018	25.05, 20.48	L8	64	14.2	4.9	48.1	51.5
White Sands	10 October 2018	32.92, −106.35	L8	58	8.3	0.5	40.5	43.6
**Barreal Blanco**	12 March 2019	−31.86, −69.45	L8	45	1.0	6.3	35.5	43.1
Gobabeb	4 February 2019	−23.6, 15.12	S2B	17	3.9	4.9	35.3	28.3
Gobi Desert	11 February 2019	40.13, 94.34	S2A	18	10.4	7.4	56.5	57.3
Libya 4	4 November 2018	28.55, 23.39	S2A	20	26.2	5.4	49.8	46.2
Libya 4	3 January 2019	28.55, 23.39	S2A	28	3.4	5.7	58.1	54.8
Baotou	13 February 2019	40.85, 109.63	S2A	25	7.0	7.5	59.8	57.3
Atacama, Chile	10 March 2019	−22.49, −69.11	S2A	37	3.3	6.1	27.6	33.8
**Barreal Blanco**	12 March 2019	−32.86, −69.45	S2B	36	1.0	9.3	35.5	41.1

**Table 6:** Cross-calibration combined difference statistics (%).

Statistic	Coastal Aerosol	Blue	Green	Red	NIR
	(∼443 nm)	(∼482 nm)	(∼562 nm)	(∼655 nm)	(∼865 nm)
Difference (%)	0.50	0.54	−0.08	−0.04	−0.31
Standard Deviation	2.77	2.13	1.86	1.92	1.76

**Table 7:** Overview: Spectral in-orbit validation using the Oxygen A band absorption. Shifts in [nm] calculated as average over all cross-track elements relative to the nominal band wavelengths for the Gobabeb scene DT2019020405. “Lab. cal” refers to the pre-launch laboratory calibration, “Vic. cal.” to the vicarious in-orbit calibration.

Band	Lab. cal.		Vic. cal.	
	No Smile corr.	Smile corr.	No Smile corr.	Smile corr.
141 (∼760 nm)	−0.52	−0.46	0.19	0.26
143 (∼765 nm)	−0.52	−0.46	−0.09	−0.02
146 (∼773 nm)	−0.53	−0.45	−0.37	−0.29

**Table 8:** Spectral smile in-orbit validation. Center wavelengths given in [nm]; “Nominal” refers to the in-orbit calibration and “Validation” to the results derived for the Gobabeb scene DT2019020405 with smile correction, but no destriping.

Band		Cross-Track Element				
		10	256	512	768	1014
141	Nominal	761.31	761.13	760.63	760.46	759.81
	Validation	760.51	760.41	760.76	760.46	760.11
143	Nominal	765.81	765.57	765.32	765.13	765.05
	Validation	765.45	765.35	765.70	765.40	765.04
146	Nominal	773.45	773.18	772.83	772.53	771.90
	Validation	773.18	773.08	773.43	773.13	772.78

**Table 9:** Linear root mean square error with respect to reference scenes.

${\mathrm{RMSE}}_{X}$ (Easting)	${\mathrm{RMSE}}_{Y}$ (Northing)
21.0 ± 5.9 m	21.4 ± 6.0 m

**Table 10:** RadcalNET sites used for BOA surface reflectance study with their corresponding coordinates, extension and surface reflectance variability across site [130,131,132].

RadcalNet Site	Coordinates (°)		Extension	ρ Site Variability
Name	lon	lat	(km × km)	(%)
Railroad Valley (RVUS)	38.497	−115.690	1.0	1.5
Gobabeb (GONA)	15.120	−23.600	0.5	3

**Table 11:** Pixel defects on the DESIS chip (unbinned readout).

Manufacturing Defects		
Band	Cross-Track Elements	Defect Type
1	1–280, 1008–1024	Manufacturing Defect
2	1–229, 1016–1024	
3	1–150, 1021–1024	
4	1–150	
5	1–42	
6	1–30	
7	1–30	
8	140	Dead Pixel
9	140	
19	217	
23	277, 887	
24	103, 733	
34	802, 803	
50	728	
53	99	
54	99	
57	37	
69	836	
70	836	
220	619	
221	619	

**Table 12:** Performance (%) for solar panels detection.

Product	Overall Accuracy	False Positives
Multispectral	34.39	0.01
Fused product	92.68	0.10

## Abbreviations

Main abbreviations in this manuscript are given below:

AOT	Aerosol Optical Thickness
BOA	Bottom-Of-Atmosphere
BRDF	Bi-directional Reflectance Distribution Function
CDOM	Colored Dissolved Organic Matter
CP	Control Points
CMOS	Complementary metal-oxide-semiconductor
DC	Dark Current
DCT	Discrete Cosine Transform
DM	Detector Map
DN	Digital Number
DDV	Dark Dense Vegetation
DESIS	DLR Earth Sensing Imaging Spectrometer
DLR	German Aerospace Center
DEM	Digital Elevation Model
DSM	Digital Surface Model
EnMAP	Environmental Mapping and Analysis Program
ES	Edge Slope
GCP	Ground Control Points
GSD	Ground Sampling Distance
ISS	International Space Station
MTF	Modular Transfer Function
MUSES	Multi-User-System for Earth Sensing
NIR	Near Infrared
PICS	CEOS Pseudo-Invariant Calibration Sites
RCN	RadCalNet
ROI	Region Of Interest
SA	Spectral Angle
SNR	Signal-to-Noise Ratio
SRF	Spectral Response Function
SRTM	Shuttle Radar Topography Mission
TBE	Teledyne Brown Engineering
TOA	Top-Of-Atmosphere
TSM	Total Suspended Matter
VNIR	Visible Near Infrared
WV	Water Vapor
